# Combinatorial Complexity and Compositional Drift in Protein Interaction Networks

**DOI:** 10.1371/journal.pone.0032032

**Published:** 2012-03-08

**Authors:** Eric J. Deeds, Jean Krivine, Jérôme Feret, Vincent Danos, Walter Fontana

**Affiliations:** 1 Center for Bioinformatics and Department of Molecular Biosciences, University of Kansas, Lawrence, Kansas, United States of America; 2 Laboratoire PPS de l'Université Paris 7 and CNRS, F-75230 Paris, France; 3 Laboratoire d'Informatique de l'École normale supérieure, INRIA, ÉNS, and CNRS, F-75230 Paris, France; 4 School of Informatics, University of Edinburgh, Edinburgh, United Kingdom; 5 Department of Systems Biology, Harvard Medical School, Boston, Massachusetts, United States of America; Hospital for Sick Children, Canada

## Abstract

The assembly of molecular machines and transient signaling complexes does not typically occur under circumstances in which the appropriate proteins are isolated from all others present in the cell. Rather, assembly must proceed in the context of large-scale protein-protein interaction (PPI) networks that are characterized both by conflict and combinatorial complexity. Conflict refers to the fact that protein interfaces can often bind many different partners in a mutually exclusive way, while combinatorial complexity refers to the explosion in the number of distinct complexes that can be formed by a network of binding possibilities. Using computational models, we explore the consequences of these characteristics for the global dynamics of a PPI network based on highly curated yeast two-hybrid data. The limited molecular context represented in this data-type translates formally into an assumption of independent binding sites for each protein. The challenge of avoiding the explicit enumeration of the astronomically many possibilities for complex formation is met by a rule-based approach to kinetic modeling. Despite imposing global biophysical constraints, we find that initially identical simulations rapidly diverge in the space of molecular possibilities, eventually sampling disjoint sets of large complexes. We refer to this phenomenon as “compositional drift”. Since interaction data in PPI networks lack detailed information about geometric and biological constraints, our study does not represent a quantitative description of cellular dynamics. Rather, our work brings to light a fundamental problem (the control of compositional drift) that must be solved by mechanisms of assembly in the context of large networks. In cases where drift is not (or cannot be) completely controlled by the cell, this phenomenon could constitute a novel source of phenotypic heterogeneity in cell populations.

## Introduction

A large fraction of current data in molecular biology has been derived from the collation and curation of predominantly static types of data, such as genomic sequences and protein structures. However, at increasing rate, proteomic high-throughput methods, such as yeast two-hybrid assays, protein complementation assays, affinity purification with mass spectrometry, peptide phage display, and protein microarrays are yielding data about protein-protein interactions (PPI) whose significance resides in the system behavior they collectively generate [1]–[5]. In conjunction with more thorough biochemical measurements, these interaction data yield mechanistic statements ranging from less detailed, as in “*a phosphoepitope of EGFR binds strongly to the SH2/PTB domains of Grb2, Nck1, PI3K* 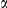 *and weakly to the SH2 domains of Grb10, Grb7, Nck2, Shp1*”, to more detailed, as in “*axin1 binds a region in the armadillo repeat of* 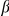 *-catenin, if* 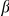 *-catenin is unphosphorylated at certain N-terminal residues.*” Unlike structural and genomic data types (“molecular nouns”), interaction fragments of this kind (“molecular verbs”) are fundamentally about process, and their broader meaning resides in the dynamic behavior of the large networks they generate.

High-throughput assays, such as yeast two-hybrid (Y2H), typically probe for pairwise binding between proteins in a highly impoverished context, lacking excluded volume and other effects that might influence interactions when the proteins tested are bound to multiple others [2], [6]. Interaction data of this kind are often rendered as a large graph in which nodes represent proteins and edges correspond to pairwise binding interactions reported by the assay. These graphs have been shown to possess statistical properties, such as bow-tie structure [7], [8], approximately scale-free degree distributions [9] and small-world characteristics [10]. Yet, unlike road networks, the edges in PPI networks do not represent persistent physical connections between nodes, but rather summarize interaction *possibilities* that must be realized through physical binding events. The cumulative effect of such events results in a distribution of protein complexes that ultimately determines cellular behavior. Significant properties of PPI networks may therefore become apparent only by studying the behavior they induce in a population of proteins, which requires the development and analysis of dynamic models.

**Figure 1:**
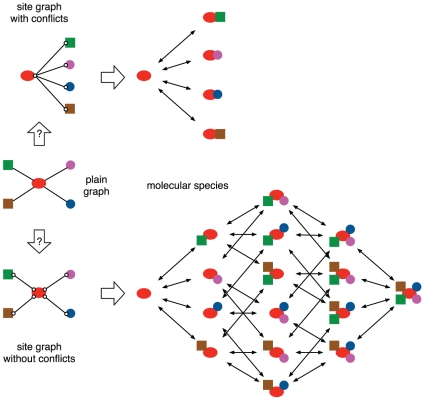
Binding surfaces and complex formation. Center: The traditional plain graph representation of a PPI network represents the binding capabilities of a hub protein (red) through several incident edges. The diversity of molecular species generated by these potential interactions depends on the extent to which they compete for binding surfaces (white circles), to which we refer as “sites”. These conflicts are best represented as a “site graph”, derived from a domain-level resolution of protein-protein interactions. We depict two extreme cases. Top: All interaction partners compete for the same site. Bottom: All interactions occur at different sites and are mutually compatible. In the language we deploy to represent processes based on protein-protein interactions, a site denotes a distinct interaction capability. A comparison between the scenarios depicted at the top and the bottom illustrates how combinatorial complexity is affected by binding conflicts.

The first problem in constructing a dynamic model from raw PPI data is the lack of sufficient structural information. For instance, it is a priori unclear whether a “hub” protein with many interactions in the PPI network employs just one surface or many surfaces. As Figure 1 indicates, the set of complexes in which such a protein could participate depends on this information, since it allows the distinction between individual interactions that are mutually compatible and those that are mutually exclusive. The Structural Interaction Network (SIN) of yeast [11] is a dataset that provides this needed level of resolution.

It is often assumed that the various domains of a protein interact independently of one another; that is, the capacity of a protein's domain *A* to bind its various partners is independent of the binding state of domain *B* on that same protein. While such an assumption represents an extreme case, so too does the assumption that domain *A* can bind only when domain *B* is unbound, or an assumption that posits strict allosteric correlations among binding partners. In the absence of systematic and readily accessible knowledge about steric and allosteric constraints in large-scale protein interaction networks, we consider the case of complete independence (subject to general biophysical constraints discussed below) as a useful “what-if” scenario against which to assess the significance of departures from independence.

The independence assumption creates a major challenge for making and running a model of a PPI network: the number of possible complexes (i.e. unique molecular species) that the network can generate increases exponentially as the network grows, reaching astronomical numbers for biologically reasonable networks [12], [13]. This situation necessitates an implicit representation of interactions as *local rules*, since models based on the explicit representation of all molecular possibilities, such as systems of differential equations, are entirely unfeasible. In recent years, we and others have developed appropriate tools for the representation and simulation of combinatorially complex systems of this kind [14]–[20].

In this contribution, we join two critical components–a suitable dataset and a modeling methodology–to simulate a large slice of the SIN network. By taking into account the inherent combinatorial complexity of the network, we extend pioneering calculations by Maslov and Ispolatov [21]. We consider neither post-translational modifications nor synthesis and degradation processes, as the available SIN data is exclusively about binding. Our simulated systems therefore reach thermodynamic equilibrium, although we shall see that this seemingly peaceful picture does not do justice to the microscopic dynamics. The main motivation for studying a highly abstracted and thus somewhat fictitious biochemical system is threefold. First, the image of a causally unconstrained network of possibilities, as conjured up by Y2H, has been taken seriously enough to attract extensive statistical investigation [22]–[25] of its structural properties. It seems warranted, therefore, to complement such studies with an eye on the dynamical properties implied by a similarly unconstrained interpretation of Y2H data. Second, the dynamic behavior of such a network serves as a null model to understand the need for and the consequences of curtailing independence through, for example, post-translational modification and allosteric interaction. In other words, studying the dynamics of the null model identifies a type of problem that specific causal constraints might have evolved to address, as we argue in the “Discussion” section. Third, the simulation of SIN dynamics represents a challenging test case illustrating a number of concepts underlying recent rule-based modeling methodologies [13]–[15], [17], [20] that are applicable to more general situations.

## Methods

### Interaction network data

As mentioned above, in order to provide a more structural picture of protein interaction networks, Kim *et al.* [11] combined raw interaction data from high-throughput experiments with data regarding domain-domain interactions in solved protein structures. This “Structural Interaction Network”–or SIN–associates a surface or domain of a protein with each interaction, converting the traditional flat graph into a site graph or domain-level interaction network of the type shown in Figure 1. We obtained the original SIN directly from the authors. It consists of 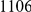 distinct proteins and 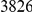 specific pairwise interactions (edges).

Two proteins belong to the same graph component if there is a path of edges connecting them. The SIN has several such components. The largest (or “giant”) component consists of 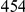 proteins and 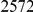 interactions. The giant component contains 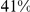 of the nodes in the graph, but includes 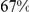 of its interactions. It therefore exhibits a significantly higher edge density (i.e. the fraction of possible edges present), 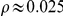, than the rest of the graph, 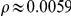. The second-largest component in the SIN has only 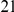 proteins and most of the other components consist of only 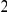 proteins, representing isolated dimerizations. Current computational power precludes simulation of the dynamics of the entire SIN. Since the giant component contains a majority of the SIN interactions (and most of the interesting structure), we focussed on this part of the graph.

**Figure 2:**
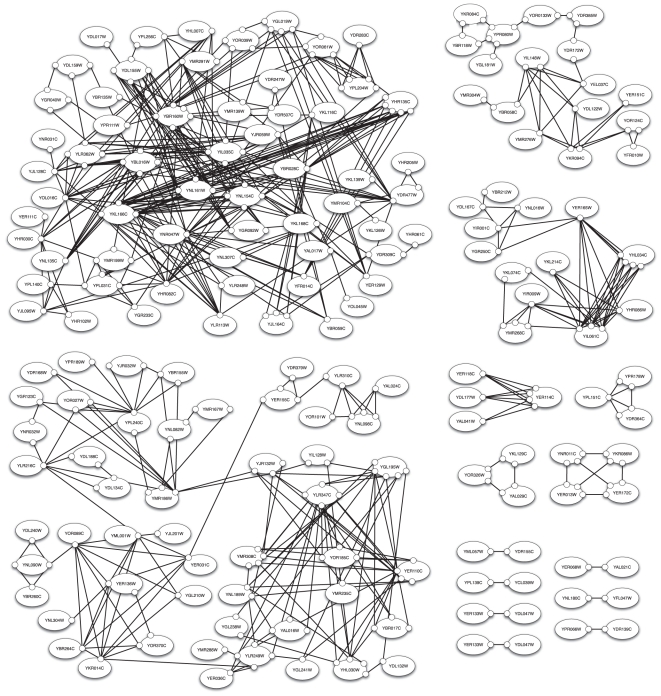
The network subject of this paper. The graph of proteins, sites and interactions found in the cytoplasmic portion of the Structural Interaction Network (cSIN), as compiled by Kim et al [11]. The cSIN displays interactions at the level of domains or binding surfaces, making explicit which interactions compete for the same binding site. We refer to such a graph as a site graph. Its nodes are proteins (ovals), which are sets of sites (small circles on the ovals). Sites, rather than proteins, anchor the edges of this graph.

Data on subcellular localization and copy number were obtained from the “yeastgfp database” described in [26], [27]. This database contains information for about 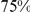 of the proteins in the SIN. Using this data, we determined compartment-specific subgraphs of the SIN, consisting of only those proteins and their interactions that co-occur in the same compartment. These subgraphs exclude proteins that are found in a compartment but do not interact with any of the other proteins in that compartment, since such proteins could not participate in any kind of binding dynamics in our simulations. The cytoplasmic subgraph of the SIN consists of 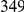 proteins and 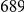 reactions. If we restrict ourselves to just the cytoplasmic subgraph of the giant component (which contains 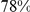 of the interactions), we obtain a system with 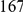 proteins and 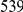 reactions, shown in Figure 2, which defines the network we simulated. We call this cytoplasmic subgraph of the giant component of the SIN the “cytoplasmic SIN” or cSIN for short.

Although homomeric interactions (i.e. a protein interacting with itself on some site) are certainly common, no such interactions have been characterized for this particular set of proteins: the Saccharomyces Genome Database (SGD, http://www.yeastgenome.org) lists no homomeric physical interactions for proteins in the cSIN.

Copy numbers were assigned to each of these 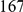 proteins directly from the yeastgfp data [26]. In those cases where a protein is listed as existing in more than one compartment, assignment of a copy number to the cytoplasm becomes ambiguous. In the absence of data regarding the relative concentration of a given protein among compartments, we assumed that its concentration in each compartment is approximately equal. Since the cytoplasm represents the majority of the cell's volume (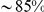 [28]), we simply assigned all copies of that protein to the cytoplasm. With this initial condition, the total number of individual protein agents present in each of our simulations was 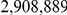.

The localization and copy number data we used are based on measurements in asynchronous populations of cells [26], [27]. Our simulations do not take into account variations in copy number that might occur during the cell cycle [29]–[33]. However, only 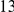 of the 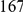 cSIN proteins exhibit strongly significant variations in expression level over the cell cycle, in the sense of being among the top 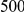 scoring yeast genes in a recent analysis [32]. Although changes in copy number during the cell cycle can clearly influence the types of complexes present in the cell [33], we leave consideration of these effects to future work.

A file with the complete set of interaction rules of the cSIN together with the initial condition is available as Supporting Information S2.

### Executable representation of the interaction network

**Figure 3:**
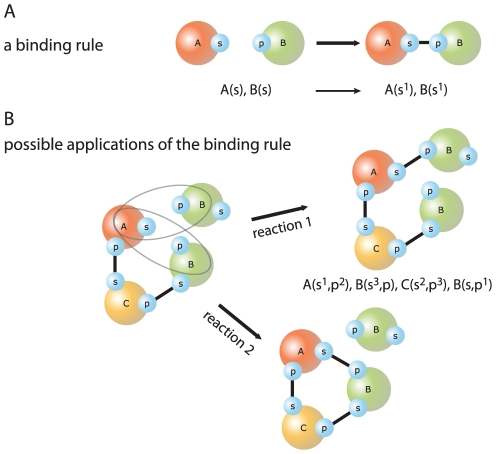
Kappa rules. **A:** A rule expresses a local mechanistic statement (of empirical or hypothetical origin) about a protein-protein interaction in terms of a rewrite directive plus a rate constant (not shown). The left hand side (LHS) of the rule consists of partially specified protein agents, and represents the contextual information necessary for identifying reaction instances that proceed according to the rule. The right hand side (RHS) expresses the actions that may occur when the conditions specified on the LHS are met in a reaction mixture. In this case, the rule specifies a binding action. Site graphs are represented in a simple syntax, explicated in Figure 1 of Supporting Information S1. **B:** The rule in panel A can match the shown sample mixture of molecular species in two ways, giving rise to two possible reactions with different outcomes. Because of their local nature, Kappa-rules may apply in both a unimolecular and bimolecular situation. In general, such rules are given two rate constants (a first-order and a second-order constant), and the simulator will automatically generate the appropriate stochastic kinetics. However, in the present paper, global constraints prevent this ambiguity at the outset and the rules of the cSIN therefore necessitate only one rate constant (bimolecular for association and unimolecular for dissociation).

A graph of *prima facie* independent binding interactions of the kind shown in Figure 2 permits a huge number of possible complexes (which we estimate in the “Results” section below). The vast number of possible molecular species rules out any modeling approach that requires their *a priori* enumeration. The only feasible simulation approach is one that replaces reactions between molecules with *local rules* that only specify which state modifications occur (in our case association or dissociation) and the sites on which these modifications depend (Figure 3). Reactions, on the other hand, must completely specify the binding state of each participating protein. A large set of reactions might express the same fundamental event in all of its possible contexts, whereas a rule can represent this entire family of reactions by specifying only the minimal context necessary for the event to occur. Rules can thus capture non-covalent association and dissociation of proteins or, more generally, post-translational modifications in a way that respects, as and when appropriate, the local quality of these interactions.

In representing and executing the cSIN, we follow our specification and implementation of a rule-based language, known as Kappa [14], [17], [18], [34]–[37], which is conceptually related to the Biological Network Generator Language (BNGL) [15], [16], [19], [20]; see section 1 of Supporting Information S1. Rules that stipulate no other context than the domains involved in a binding or unbinding interaction between two proteins correspond exactly to the edges in the cSIN. We convert each edge into a pair of Kappa rules of the kind
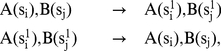
(1)representing a binding (or unbinding) interaction between the 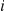
th site of protein A and the 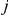
th site of protein B. The superscript expresses a bond between the sites. For example:
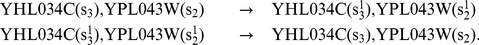
(2)Such rules of local interaction are then applied to a computational mixture consisting of a large graph whose nodes represent individual proteins and whose connected components represent protein complexes, much like the application of the rule in panel A of Figure 3 to the two-molecule mixture in panel B. Rule applications occur with probabilities in accordance with stochastic chemical kinetics, giving rise to a continuous-time Markov process implemented as detailed in [18], [19], [38] and summarized in Supporting Information S1. At the start of a simulation, each protein is present with a number of copies derived from the previously mentioned empirical data, resulting in a total of 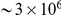 individual protein agents.

### Affinities

In order to simulate the dynamics of a PPI network, we must assign to each (independent) binding reaction both an on-rate 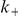 (the rate constant for the first type of rule in equation 2) and an off-rate 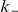 (the rate constant for the second type of rule in equation 2). The dissociation constant, 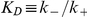, is a measure of the strength or affinity of the corresponding interaction. Since high-throughput PPI experiments do not provide information about interaction strengths, we consider below three broad cases. The conversion into rate constants is discussed in the subsequent section.

#### Uniform affinities

Even when all of the binding reactions in the network have the same affinity, the question remains as to exactly *which* universal affinity to choose. The protein interaction strengths found in the PINT database exhibit an average affinity equivalent to a 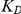 of 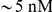 [21], [39]. Since these interactions are obtained for a wide variety of proteins (many of which are not found in yeast and many of which represent mutated interaction pairs) and under a wide range of conditions (i.e. pH values and temperatures that are not necessarily characteristic of the yeast cytoplasm), it is difficult to interpret what this average value might mean for the cSIN. We therefore chose to look at a variety of 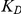 values: 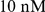, 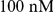 and 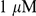. The 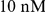 case represents a set of fairly strong interactions (close to the average in PINT [21], [39]) and the 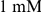 case represents a set of fairly weak interactions.

#### Concentration-based affinities (“equal saturation”)

Even for strong interaction strengths (e.g. 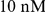), the log-normal distribution of protein concentrations observed within the cell causes reactions to operate at widely differing saturation levels. For instance, an interaction between two proteins at a concentration of 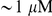 will be highly saturated when assuming a 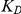 of 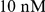, while an interaction between two other proteins present at 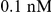 will not be saturated at all. Following Maslov and Ispolatov [21], we consider a case in which each reaction in the network operates at approximately the same level of saturation. Consequently, we require the reaction affinities to vary with the (initial) reactant concentration as
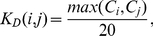
(3)where 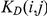 is the dissociation constant of binding between proteins 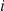 and 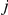, and 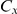 denotes the total concentration of protein 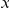 (obtained from experiment [40]). This method ensures that the overall binding saturation is essentially constant across reactions in the network when physiological concentrations are employed. The set of 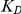
's obtained from equation 3 are log-normally distributed [40], and has recently been shown to represent a biologically and biophysically realistic case [41], [42].

#### Structure-based affinities

We can estimate binding affinities directly from the protein structures on which the interaction network is based [11]. Several studies have noted that the change in solvent-accessible, non-polar surface area that occurs on binding, 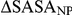, is linearly related to the free energy of association [43], [44]. To make use of this fact, we first re-constructed (as detailed in section 8.2 of Supporting Information S1) the PPI network on the basis of the domain-domain interaction structures referenced in the most recent release of iPfam. We call this network the “cSIN2.” For each interaction in the cSIN2, we used the software package POPS [45] to determine the average 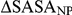 taken over all the instances of that particular domain-domain interaction in iPfam. Using a recently published data set [44], we performed a linear regression to map 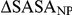 into the corresponding free energy of binding 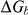. Although the correlation in this case is certainly not perfect (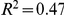, see Figure 11 of Supporting Information S1), the resulting equation provided us at least with a rough estimate of 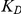 (as 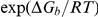) for each interaction in the cSIN2.

### Rate constants

We next describe the convesrion of affinities into on- and off-rates. Let 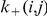 denote the rate constant of the binding reaction between proteins 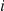 and 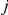 (on-rate) and let 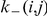 denote the dissociation rate constant for that bond (off-rate). Since 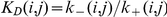 only constrains the ratio of the rates, we can choose either the on- or the off-rate arbitrarily and still satisfy a specified reaction affinity.

**Figure 4:**
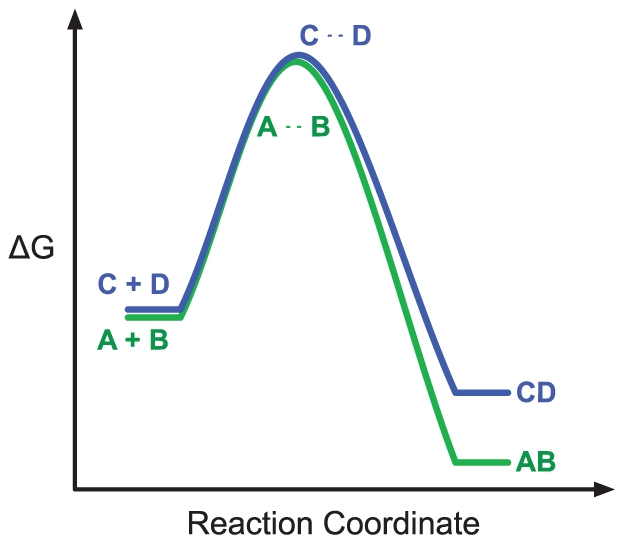
Schematic free energy landscape. The schematic shows the free energy landscape for a case in which differences in affinities are entirely represented by differences in off-rates. Here we have two different binding reactions: A binds B and C binds D. “A+B” and “C+D” represent the unbound states on the far left of the schematic reaction coordinate; the unbound states in this case have roughly the same free energy. The transitions states (represented by “A 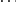 B” and “C 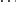 D”) also have approximately the same free energy; the change in free energy from the unbound state to the transition state is identical in both cases (giving identical values of 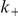). However, the bound states (“AB” and “CD”) exhibit very different free energies, and the difference in free energy change between the transition state and the bound state results in a much higher value of 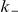 for the C–D binding reaction compared to the A–B binding reaction.

In the present work, we constrain the on-rate to *always* have the same value, regardless of the 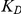. When all reactions in the network have the same affinity, varying the global affinity (e.g. from 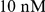 to 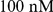) thus amounts to varying the probability that bonds will be broken once they are formed. This means that the relative change in free energy between the unbound state and the binding transition state is the same for all reacting pairs; all that changes is the free energy of the bound state, as illustrated schematically in Figure 4. It appears reasonable [41], [42] that much of the differences in binding free energies across the network are due to differences in relative hydrophobicity. However, in cases where the transition state free energy includes significant electrostatic contributions, one might expect significant variance in both on- and off-rates [46].

Equipped with deterministic rate constants 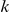 for each of our reactions, we convert these into stochastic rate parameters 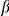. A dimensional argument suggests that for a unimolecular unbinding reaction 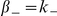 in units of s^
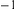
^, while for a bimolecular binding reaction
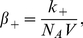
(4)in units of molecule^
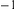
^s^
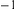
^, where 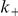 is the deterministic rate constant in units of M^
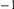
^s^
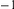
^, 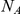 is Avogadro's constant and 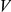 is the volume of the system in liters. Microscopically, the inverse volume dependence arises from converting the “collision volume” swept out by a moving molecule into a probability through division by the volume available to an encounter, i.e. the volume of the system [38]. A unimolecular reaction has no collision volume and therefore its stochastic rate is independent of the system volume.

Since the protein copy numbers used in our simulations were obtained for haploid yeast cells, we approximate the volume to be 42 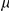
m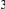, or 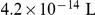 [47]. We set the on-rate 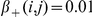 for all 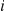, 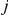 in the network, which corresponds, by equation 4, to a deterministic on-rate of 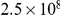 M^
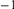
^s^
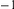
^. Given the absence of empirical measurements, the value of 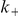 (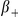) is not meant to be realistic. Interactions driven purely by hydrophobicity could have values 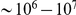 M^
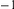
^s^
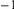
^ [48]. The time scales discussed in the “Results” section are estimated assuming this range of on-rates, but it is important to note that the actual on-rates observed in a living system might differ significantly. Hence, for our simulations, the unit of time is essentially arbitrary.

### Preventing polymerization

A local cSIN rule like equation 2 specifies the binding between specific domains of proteins A and B, without, however, specifying whether A and B are members of the same or distinct complexes. In the first case the interaction is intramolecular; in the second case it is intermolecular (Figure 3). When the underlying network site graph contains proper cycles (i.e. paths that start and end on the same protein node without touching a site twice), this ambiguity results in infinitely many possible rings and polymers. Without further constraints, mass action would lead to a prevalence of long polymers, but aside from cytoskeletal proteins (such as actin and tubulin) or prions there is no empirical information suggesting that proteins generally form non-covalent polymer chains. In our simulations we must, therefore, prevent or curb polymerization. We achieve this by employing *global* constraints, that is, constraints that are not expressed directly as executable rules, but as filters applied by the simulator at runtime. We implemented two scenarios that correspond to distinct structural interpretations of network cycles, which we summarize next. A detailed exposition can be found in sections 6 and 7 of Supporting Information S1.

#### The “stable rings” (SR) scenario

We might imagine that the open chain 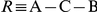 (which, in the more precise notation of our formalism, reads 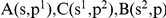) is structurally sufficiently constrained to readily form a cyclical complex by *intra*molecular binding between A and B. In this rationale, there is not enough physical room in 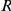 to accommodate another B in an *inter*molecular reaction with A. We refer to this scenario as “stable rings” (SR): In this case the binding site on A is assumed to be naturally occluded by the B already bound to C. In the SR scenario, ring-like structures are highly stable [49] and form *immediately* whenever intramolecular ring closure is possible. A thermodynamic justification of this scenario is discussed in section 6.1 of Supporting Information S1. Polymerization is thus prevented by the formation of stable rings and a constraint enforcing the excluded volume implied by the SR scenario (Figure 5 of Supporting Information S1).

**Figure 5:**
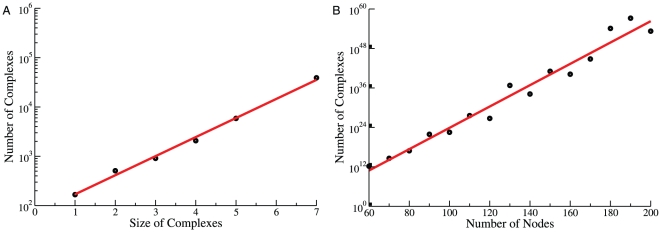
Combinatorial complexity of the cSIN. **A:** Panel A reports the number of unique complexes that could be produced by the cSIN as a function of complex size using brute force enumeration. As described in the text, complexes that contain more than one copy of a particular protein are discarded, since they could correspond to polymers. Given that the NR constraint allows for multiple copies of a protein to enter a complex in certain situations (see section 7.1 of Supporting Information S1), the numbers displayed here represent a lower bound on the number of unique complexes for the NR constraint. The red line represents an exponential regression of the data, with 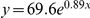. **B:** Panel B reports the estimated combinatorial complexity of cSIN-like acyclic networks as a function of network size, using the procedure described in section 3 of Supporting Information S1. Each point represents an average over 10 independently generated model networks with the same edge density as the cSIN. The red line depicts an exponential regression with 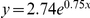.

#### The “no rings” (NR) scenario

**Figure 6:**
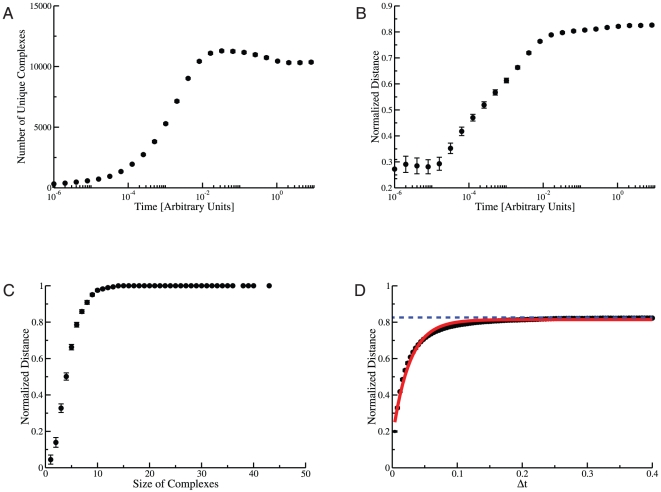
Dynamic diversity of the cSIN in yeast cells. **A:** The graph reports the number of unique complexes actually present in a simulated system (“cell”) as a function of time. Each point represents an average over 15 independent simulations. In all panels of this figure, the error bars represent approximately 95% confidence intervals. **B:** The normalized distance between the complement of complexes (“complexomes”) generated by individual simulations is shown as a function of time. Each point is an average over all unique comparisons between 15 independent simulations. Using the parameters described in the text, the separation between steady states reaches 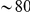
% of the maximal distance. **C:** The stationary distance between cells is shown as a function of complex size, averaged over all of the unique comparisons between 15 independent simulations. The complexomes of cells are nearly identical with regard to small complexes, due to fewer combinational possibilities and the high relative abundance of small complexes (see Figure 7 below). However, complexomes differ dramatically for large complexes. This is the case for all combinations of parameters and ring closure scenarios we have tested (see below and Supporting Information S1). Since other parameter sets do not substantially change the relationship shown here, much of the difference in inter-cell distances for these parameter sets derives from how heavily the dynamics sample large complexes. **D:** The distance between a cell at time 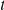 and the same cell at time 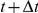 is shown as a function of 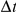. The first time point 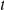 is taken after cells have reached steady state (in this case, 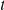 = 2, see panels A and B). The blue line denotes the average inter-cell distance at steady state, taken from the last time point in panel A above. The red curve represents an exponential fit to the relaxation, with 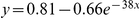.

Many steric constraints other than direct occlusion of A's binding site for B might prevent the addition of a second B to 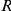. We subsume these alternative geometries under the “no rings” (or NR) scenario. The NR scenario introduces a syntactical filter that simply prevents at runtime any form of polymerization by *fiat*, as detailed in Figure 6 and section 7.1 of Supporting Information S1.

Neither the SR case nor the NR case is likely to represent the reality of complex formation in the cell. Some of the cycles in the contact map of the cSIN might represent SR complexes, others might follow the NR scenario or perhaps even give rise to polymers of limited size.

We assessed the validity of the cSIN and the soundness of our model by comparing our computational mixtures of complexes with Affinity Purification-Mass Spectrometry (AP-MS) experiments (see section 9 of Supporting Information S1). In discussing the computational results, we focus on the NR scenario since it provides slightly better overlap with experimental data.

## Results

### Estimating the Number of Reachable Molecular Species

The number of distinct molecular species–the “reachable complexes” or “reachables” for short–that can, in principle, be generated with the interactions listed in the cSIN conveys a sense for the fraction of possibilities that a population of protein agents can access at any one time.

If an interaction network does not give rise to cyclical subgraphs, the set of reachables can be enumerated. If cycles are present, as is the case in the cSIN, the set of reachables, absent any constraints, is infinite due to polymerization. The cSIN contains many proper cycles (see Figure 2), which motivated the SR and NR scenarios described above. Since these constraints are not expressed as Kappa rules, but rather enforced at runtime, we were unable to compute the possibilities inherent in the cSIN other than by brute force enumeration stratified by complex size, as reported below. This strategy is feasible only up to a modest size. However, we can estimate the combinatorial complexity of the cSIN by constructing artificial *acyclic* interaction graphs with an edge density that matches the cSIN and for which we can count the number of complexes.

#### Direct Enumeration by complex size

The cSIN consists of 167 distinct proteins, and thus 167 unique monomers, and 539 dimers, since every interaction in the network can form a unique dimer. Starting from the set of dimers, we can create a set of trimers by taking a free site in every such dimer and adding a possible binding partner to form a trimer. Because of cycles in the contact map, such a procedure could easily produce multiple copies of the same complex; for instance, adding a C to the B of an A- B dimer produces the same A- B- C trimer as adding an A to the B of a B- C dimer. To avoid overcounting, we simply check for each new complex whether it has already been found and, if it has, we discard it. We prevent polymeric complexes by simply requiring that no agent type occurs twice in the same complex. This is a stricter criterion than the no-polymerization constraint of the NR scenario mentioned above. As such our counts constitute lower bounds for the NR case. Starting with the set of unique trimers, the set of tetramers is calculated in much the same way. We iterate this procedure up to complexes of size 7. The results are shown in Figure 5A. Truncating the enumeration at this point results in nearly 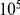 unique molecular species. Unfortunately, for complexes of size 8 or larger the computational cost of checking for duplicates exceeds current computational resources. Despite this limitation, brute-force enumeration up to size 7 indicates that the cSIN is likely to generate a very large number of possible unique complexes.

#### Complexes in Random Acyclic Graphs

We construct random acyclic interaction graphs (RAGs) with varying number 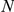 of nodes but a fixed cSIN edge density 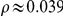 and compute the number of possible complexes, as detailed in section 4 of Supporting Information S1. Each point in Figure 5B reports the average number from 10 independently generated RAGs with a given 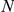. Although we cannot give a tight estimate for the cSIN, we conclude from Figure 5B that the number of possible unique cSIN complexes is in the range of 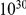 to 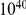, which is much larger than the total number of proteins present in any given yeast cell. This approach assumes, however, that all possible complexes can be physically realized. In section 5 of Supporting Information S1, we describe a simple calculation to estimate the consequences that steric constraints might have on the total number of molecular species that an interaction network could form. The case we considered represents a fairly strong constraint, in which steric effects become more and more prominent as complexes get larger. Given that the surface area of a complex will tend to increase with increasing size, this might not represent the most realistic situation, but the model demonstrates that even strong steric constraints do not curtail combinatorial complexity significantly. If only 20% of complexes of a given size can be realized, the total number is still 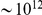, suggesting that steric constraints would have to be incredibly strong in order to reduce the number of molecular possibilities to numbers that allow their simultaneous sampling by a cell.

### Network dynamics with uniform affinities

Based on our assumptions about affinities and rate constants (Methods section), uniform affinities translate into uniform rate parameters. The case we discuss here consists in a stochastic dissociation constant 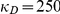 molecules (corresponding to a deterministic 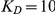 nM); a stochastic on-rate 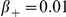 molecule^
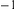
^ s^
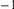
^ (corresponding to a deterministic on-rate 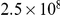 M^
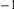
^s^
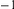
^); and a stochastic off-rate 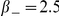 s^
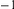
^ (corresponding to a determinsitic off-rate 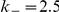 s^
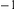
^). Results for other uniform interaction strengths are similar and are discussed in Supporting Information S1.

The number of unique molecular species present as a function of time (averaged over 15 independent simulations) is shown in Figure 6A. The system approaches a steady-state comprising around 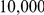 unique complexes. The approach to steady state occurs on a time scale that corresponds roughly to the equilibration of individual binding reactions. Significantly weaker interactions lead to somewhat fewer unique species, as does the SR scenario. In all cases, no single (simulated) cell contains enough unique complexes to even sample all of the 7-mer structures compatible with the network (Figure 5A), much less the set of all possible complexes. To characterize the differences between simulations, or independent “cells”, we define the set of unique complexes in a cell 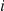 as 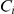 and the distance between two cells 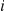 and 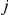 as:
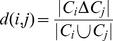
(5)where 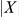 denotes the number of elements in set 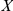 and 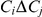 denotes the symmetric difference (i.e. the set of complexes that are either in cell 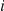 or cell 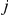, but not both). Normalizing the symmetric difference by the union 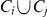 results in a 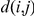 representing the probability that a particular type of complex found in either cell 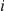 or cell 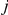 is unique to one cell or the other. Although cells start out as identical, they rapidly diverge to a distance of about 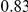, indicating that only 17% of complexes are found in both cells at steady-state (Figure 6B). Alternative distance functions, including definitions that consider differences in copy number, produce similar results (see Supporting Information S1). The exact value of the steady-state distance depends on details and parameters of the simulations: The SR scenario leads to lower distances–as low as 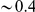 (see Supporting Information S1).

The divergence of initially identical cells in the space of possible complexes varies strongly with complex size and copy number (Figure 6C of this text and section 8 of Supporting Information S1). All cells exhibit an essentially identical repertoire of monomers, dimers and trimers, which tend to be the most common complexes. However, for complexes of size 9 or larger, cells tend to be completely distinct from one another. We generally find only a single example of any given large complex in a cell, and any particular large complex found at time 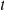 in one cell will not be found anywhere else in the population (Figure 6C). This finding is robust to changes in the affinity parameters and characterizes both the SR and NR constraints (see Supporting Information S1).

**Figure 7:**
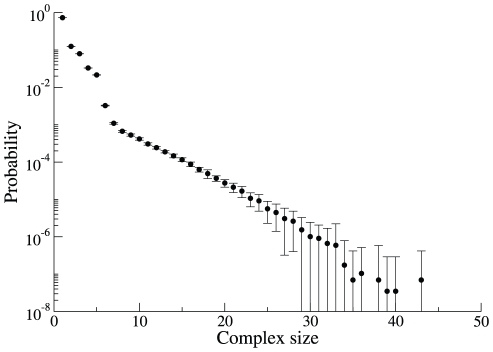
Distribution of complex sizes. The graph shows the distribution of complex sizes for NR simulations with all dissociation constants set to 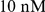. This distribution is calculated at the final time point for the simulations represented in Figure 6. The points on the graph represent the average probability of finding a complex of a certain size across 15 independent simulations. The error bars in this case are set to approximate 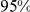 confidence intervals; for large complexes, the error bars exceed the scale for the lower bound. This is because the 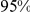 confidence intervals include 0, which cannot be displayed on the logarithmic scale of the ordinate.

Figure 7 shows the distribution of complex sizes at steady state. This distribution is derived from the same set of simulations examined in Figure 6. Small complexes (i.e. monomers and dimers) clearly dominate the distribution, with larger complexes being comparatively rare. The dominance of monomers in this case is somewhat surprising; the interactions here are fairly strong, so one would expect most proteins to participate in at least one complex. The empirical distribution of protein copy numbers, however, is approximately log-normal [40]. The most common protein in these simulations is present with over 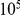 copies, while the least common protein has only 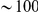 copies. Thus, certain proteins are present at much higher concentration than any of their potential binding partners, leaving many of the former as monomers. Although quite rare, the largest complexes sampled by these simulations have over 40 members.

These results suggest that each cell on its own might drift in the space of complexes. As seen in Figure 6D, the distance between a particular cell at times 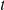 and 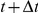 rapidly increases. For a realistic binding rate (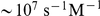) [48], the time-scale on which a cell loses memory of its former “compositional self” is 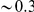 seconds. We refer to the independent sampling of a distinct and constantly varying set of complexes over time as “compositional drift”.

### Network dynamics with concentration-based affinities

**Figure 8:**
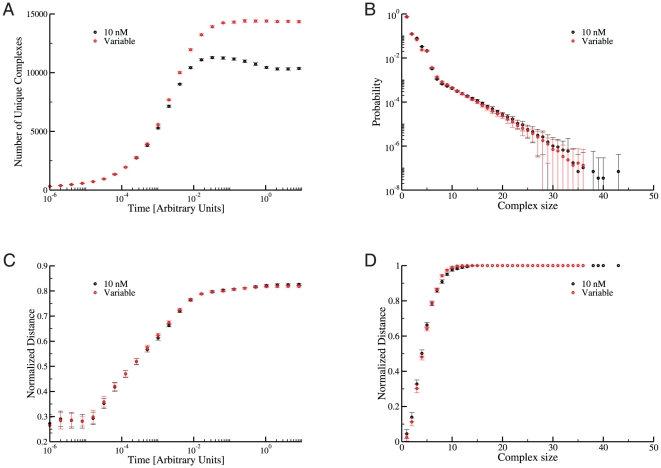
Comparison between network dynamics based on uniform affinities and concentration-basd affinities. **A:** The number of unique complexes in independent simulations as a function of time: each curve represents the average over 15 independent simulations. In this panel, as with all of the panels in this figure, the error bars represent 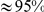 confidence intervals. Allowing interaction strengths to vary across the network produces more unique complexes at steady state (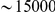 for the variable case compared to 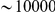 for the 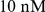 case). **B:** Comparison of the distribution of complex sizes: the distributions represent the probability of finding a complex of a particular size across the entire population of 15 simulations at the final time point in panel A. The two interaction affinity scenarios produce similar distributions, with the 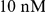 simulations sampling somewhat larger complexes. **C:** Comparison of the distance between independent simulations over time: each curve represents the average over all unique comparisons between 15 independent simulations using the distance measure defined in equation 5. As in panel B, the two scenarios produce essentially identical curves. **D:** Comparison of the distance between independent simulations as a function of complex size: each curve represents the average over all unique comparisons between 15 independent simulations at the final time point in panel A. Again, the two parameter scenarios produce essentially the same result.

We find that simulations in which 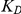
's vary across the network according to equation 3 produce results very similar to those obtained at 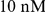 for the NR scenario. Figure 8 exhibits the appropriate comparisons. The qualitative results are the same for the SR scenario, with lower affinities leading to somewhat smaller average distances (data not shown) but still large distances for large complexes.

### Network dynamics with structure-based affinities

Proceeding as detailed in “Affinities” of the Methods section, we constructed a version of the cSIN—the cSIN2—in which each binding affinity in the network was calculated from the change in non-polar solvent-accessible surface area based on the protein structures originally used to construct the SIN itself.

**Figure 9:**
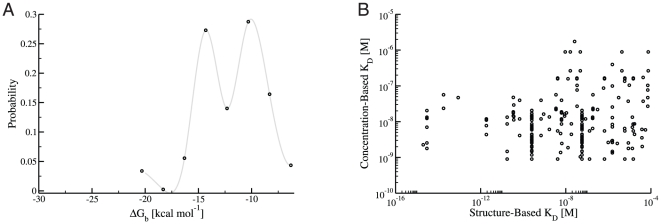
Binding free energies and dissociation constants for the cSIN2. **A:** A plot of the distribution of free energies for reactions in the cSIN2. The black circles are a histogram of the free energies; the grey line represents a smoothed version of the distribution. The average free energy is 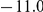 kcal mol^
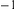
^, which corresponds to a dissociation constant of 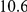 nM. **B:** This plot presents a comparison of the structure-based 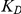
's for each edge in the cSIN2 (abscissa) and the concentration-based 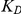
's (ordinate). For each interaction in the cSIN2 the concentration-based 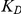 is obtained using equation 3. Despite the similarity in the average affinity in both cases (corresponding to a 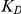 of around 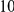 nM), the two methods produce 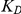 values that are very different from one another: the linear correlation produces an R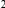 of 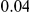.

The cSIN2 consists of 414 edges between 166 nodes. A number of edges in the original cSIN are lost in constructing the cSIN2, because some domain-domain interactions do not have representative structures in the iPfam database that are truly intermolecular, while others do not have structures where binding is strong enough (see section 8.2 of Supporting Information S1). The distribution of free energies of binding, 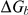, for the cSIN2 is shown in Figure 9A. It has an average of 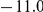 kcal mol^
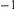
^ with a standard deviation of 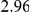 kcal mol^
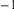
^. Interestingly, this average free energy corresponds to a dissociation constant of 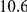 nM which is close to the average free energy seen in the PINT database [21] and used for all of the interactions in the simulations described above under the uniform rate constant scenario.

The concentration-based 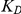 scenario (i.e. the case in which dissociation constants are derived from equation 3) yields an average affinity that is very similar to the structure-based 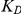
's (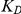
's of 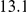 and 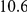 nM, respectively). However, despite the similarity in the average, the 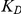 values for the structure-based affinities vary considerably across the network in a manner that appears independent from the concentration-based affinities derived from equation 3, Figure 9B.

**Figure 10:**
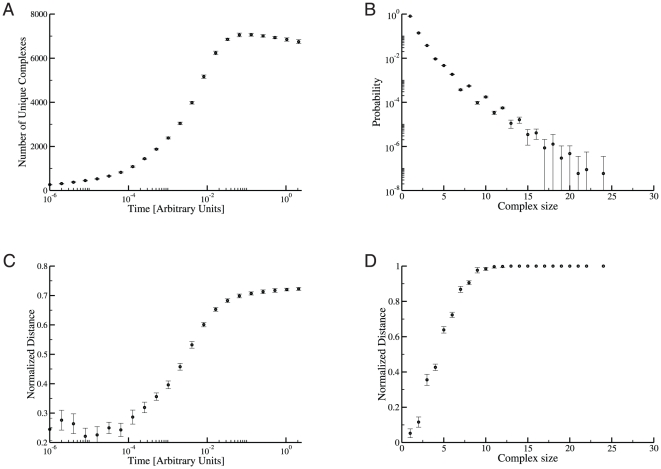
Results from NR simulations of the cSIN2. **A:** The number of unique complexes in independent simulations as a function of time: this curve represents the average over 15 independent simulations. In this panel, as with all other panels in this figure, the error bars represent 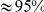 confidence intervals. The steady-state number of unique complexes is slightly smaller for the cSIN2 than the original cSIN using constant 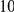 nM affinities (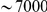 compared with 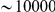). **B:** This plot shows the probability of finding a complex of a particular size across the entire population of 15 simulations at the final time point in panel A. The distribution of sizes is similar to that found for NR simulations of the original cSIN, although the complexes are, on average, somewhat smaller than those obtained from NR simulations of the cSIN at 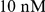. **C:** This plot displays the distance between independent simulations over time: the curve represents the average over all unique comparisons between 15 independent simulations using the distance measure defined in equation 5. The distances obtained from the cSIN2 are slightly lower than those obtained from the cSIN at 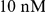 (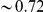 vs. 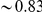). **D:** This curve represents the distance between simulations as a function of complex size, averaged over all unique comparisons between 15 independent simulations at the final time point in panel A. The overall shape of this curve is essentially identical to the 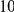 nM case for the original cSIN as displayed in Figure 5; the main difference is that the simulations based on structure-derived 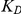
's sample somewhat smaller complexes than the original 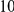 nM case.

Figure 10 summarizes the results of NR simulations of the cSIN2 using these structure-based affinities. As can be seen from Figure 10, the overall behavior of the cSIN2 is very similar to that of the original cSIN simulated with NR constraints. The cSIN2 yields somewhat lower steady-state distances than the original cSIN when simulated using 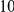 nM affinities (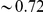 vs. 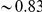) or 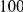 nM affinities (see Supporting Information S1), largely because the cSIN2 simulations sample somewhat fewer large complexes. SR simulations based on the cSIN2 are also very similar to the 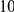 nM SR case (data not shown).

### Other results

Supporting Information S1 includes discussions of simulations using alternative distance measures (equation 5); comparisons between different uniform affinities; and the global SR scenario. The thermodynamics of ring-like protein complexes (discussed in section 6.1 of Supporting Information S1) can give rise to situations in which a particular pair of sites might not bind one another strongly enough to be detected in a high-throughput interaction screen (such as a Yeast Two-Hybrid experiment) but could nonetheless contribute dramatically to the stability of certain complexes by forming a bond to complete a ring. In Supporting Information S1 we discuss the addition of such “cryptic cycles”. All these variations leave the main observation of compositional drift intact.

## Discussion

Our simulations provide a dynamical picture of PPI networks based on a model that is respectful of their combinatorial complexity. PPI networks represent binding capabilities between proteins typically determined by an assay that yields inherently local information. Two broad components were necessary for making and running a model of a PPI network: (i) A representation of the system that can handle combinatorial complexity implicitly, since the number of possible complexes is astronomical, preventing their explicit representation. (ii) A dataset in which the interactions derived from a binding assay have been curated, and binding interactions are resolved at the level of domains or sites, allowing the distinction between interactions that are mutually compatible and those that are mutually exclusive. The first component is addressed by rule-based approaches, such as Kappa or BNGL. The second component is a suitable dataset that has been recently compiled by Kim et al [11]. We bring these two critical components together, along with protein localization, abundance data and a few biophysical assumptions, to generate a simulation of a large slice of a PPI network.

According to our simulations, systems that start from identical initial conditions diverge from one another rapidly with regard to the complexes they contain, eventually sampling different regions of the space of possible complexes. This is particularly the case for large complexes, where independent simulations tend to be essentially disjoint. Our model indicates that the complexity of such networks will result in compositional drift, even with the biophysical constraints imposed by the NR and SR scenarios. However, we consider neither post-translational modifications nor translation and degradation processes. Our systems therefore reach thermodynamic equilibrium. At equilibrium the vast space of molecular possibilities permits energetically neutral compositional drift, i.e. a never-ending change in the set of realized complexes present in a particular simulation.

The data from which our network is built has clear limitations. High-throughput methods for acquiring PPI data, such as Y2H assays, tend to have substantial false positive and false negative rates [11], [42], [50]. Curated, structure-based data sets like the SIN alleviate this drawback to some extent, but we cannot rule out the presence of fictitious edges in the cSIN network. Given that drift, especially among large complexes, is a robust feature of our simulations, it is unlikely that the ultimate removal of such edges would affect this phenomenon. Indeed, the cSIN2, which contains a slightly smaller set of interactions based on more stringent structural evidence, undergoes essentially the same level of drift as other versions of the network, indicating that inaccuracies in the underlying interaction data are unlikely to have a large influence on the overall dynamics described here (although they would have an influence on the identity of the complexes formed).

Our dynamic model does not include synthesis and degradation processes, raising the question whether limiting the time proteins persist in the cell might affect drift. High-throughput measurements of protein degradation rates [51] indicate that the average half-life of yeast proteins is around 42 minutes, with a minimum observed half-life of about 2 minutes. In our simulations, both the total number of unique complexes and their size distribution generally reach equilibrium in about one second (see, e.g., Figure 6A). Degradation processes are thus unlikely to occur at high enough rates to fundamentally influence the average size of complexes at steady-state and thus the presence of drift. However, in the SR scenario, ring-like structures are by definition so stable that they are much more likely to be removed by degradation or dilution than spontaneous dissociation. In that case, it is conceivable that degradation actually increases drift on longer timescales. Given our current computational limitations, we are unable to carry out simulations that are long enough to assess the influence of realistic synthesis and degradation rates on drift in the SR scenario.

The empirical data that define our model are also too limited and fragmentary to provide an accurate reflection of the actual geometric, kinetic, and biological constraints that determine complex formation. Indeed, large molecular machines like the ribosome and the proteasome are highly unlikely to undergo compositional drift [52]–[54]. In view of these shortcomings, what are we to make of compositional drift? At a conceptual level, our work suggests a serious problem that must be overcome in order for such complexes to assemble reliably in the cell. It is not enough for the parts of a specific supra-molecular complex to simply “fit together snugly” or bind with high affinity when independent binding sites and a large number of extraneous binding partners yield a fantastically large set of combinational possibilities that can never be exhaustively populated. Absent any further constraints, the system becomes “lost” in the vast set of possible species available to it, preventing the reliable assembly of a desired target complex.

The reduction of drift requires limiting the space of possibilities available to a PPI system. One strategy to accomplish this would be to limit the size of complexes that can form, since small complexes are well-sampled in our simulations and do not exhibit significant drift. A second strategy would be to evolve “hierarchical” assembly pathways, thus curtailing the number of accessible complexes but not necessarily their size. A simple implementation of the first strategy would be to constrain the number of sites in proteins, especially those proteins that are “hubs” in the network. Such an architecture resembles the scenario depicted at the top of Figure 1, but it does not seem to characterize the overall SIN or the cSIN studied here. Moreover, such a network architecture would not account for large macromolecular machines. A flexible inplementation of the second strategy is the use of conditional rules, where binding interactions between sites are highly sensitive to the molecular context in which they occur. There are many potential mechanisms suitable for introducing causal dependencies between binding and unbinding events: for instance, allostery and cooperativity could be employed to radically alter the binding free energy of a particular interaction in specific contexts, thus inducing the dynamics to avoid a large fraction of molecular possibilities. Post-translational modifications could also be used to create causal dependencies, provided they are deployed in such a manner as not to increase the combinatorial complexity [55].

We view compositional drift as the network analogue of the protein folding *problem*. The combinatorial explosion of possible conformational states available to the polypeptide chain raised the conundrum of how a protein can fold quickly and stably into a native structure (the so-called “Levinthal paradox”). The exploration of this problem eventually led to a framework for identifying the evolved features of free energy landscapes that ensure reliable folding of proteins [56], [57]. Likewise, the combinatorial explosion of possible molecular associations gives rise to the compositional drift problem for assembly in a network context. While there are many potential mechanisms suitable for introducing causal dependencies between binding and unbinding events, the specific deployment of these mechanisms can only be understood in light of the system-wide drift problem that they solve. In other words, compositional drift brings to light the need for complex networks to evolve particular *chemical potential landscapes* in order for assembly to proceed reliably within cells. This also raises the question, especially with regard to the many transient protein associations that can be formed during signaling, whether it is at all possible to entirely eliminate drift while reusing proteins in diverse contexts within the same cell. A certain level of compositional drift might be unavoidable, and in some situations could actually constitute an evolutionarily advantageous source of non-genetic individuality in isogenic populations.

## Supporting Information

Supporting Information S1 **This file contains a brief review of simulating Kappa models; techniques for counting complexes in acyclic graphs and for generating acyclic graphs with cSIN edge densities; and a rationale and complete description of the SR constraint.** It also contains additional results: alternative definitions of distance between simulations of the cSIN; simulations using the SR constraint; simulations using different affinities; a treatment of cryptic cycles; and a comparison with Affinity Purification/Mass Spectrometry data.
(PDF)

Supporting Information S2
**This file contains a representation of the cSIN interactions as Kappa rules.** The model is for uniform 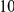 nM affinities running under the NR constraint. Due to the NR and SR constraints, the simulator executing this file is a specialized version of the open source generally available for download on www.kapplanguage.org. This specialized version is available in source and binary format from the authors.(KA)
