# Supplementary material for: Combinatorial Complexity and Compositional Drift in Protein Interaction Networks
Source: PLoS One. 2012 Mar 8;7(3):e32032. doi: 10.1371/journal.pone.0032032 (PMC3297590; doi:10.1371/journal.pone.0032032)
Supplement: Supporting Information S1 — This file contains a brief review of simulating Kappa models; techniques for counting complexes in acyclic graphs and for generating acyclic graphs with cSIN edge densities; and a rationale and complete description of the SR constraint. It also contains additional results: alternative definitions of distance between simulations of the cSIN; simulations using the SR constraint; simulations using different affinities; a treatment of cryptic cycles; and a comparison with Affinity Purification/Mass Spectrometry data. (PDF) [file pone.0032032.s001.pdf]

Supporting Information S1

for "Combinatorial complexity and compositional drift in protein interaction networks"

Eric J. Deeds[1], Jean Krivine[2], Jérôme Feret[3], Vincent Danos[4] and Walter Fontana[5]

[1]Center for Bioinformatics and Department of Molecular Biosciences, The University of Kansas, Lawrence KS 66047, USA
[2] Laboratoire PPS de l'Université Paris 7 and CNRS, F-75230 Paris Cedex 13, France
[3]Laboratoire d'Informatique de l'École normale supérieure, INRIA, ÉNS, and CNRS, 45 rue d'Ulm, F-75230 Paris Cedex 05, France
[4]School of Informatics, University of Edinburgh, Edinburgh, UK
[5]Department of Systems Biology, Harvard Medical School, 200 Longwood Avenue, Boston MA 02115, USA

Email: Eric Deeds - deeds@ku.edu; Jean Krivine - jkrivine@pps.jussieu.fr; Jerome Feret - feret@ens.fr; Vincent Danos - vdanos@inf.ed.ac.uk; Walter Fontana - walter@hms.harvard.edu;

Contents

1 A Rule-Based Modeling Framework . . . 2
1.1 Kappa . . . 2
1.2 Site graphs, contact maps, complexes, and molecular species . . . 4
1.3 Locality of rules and cyclical structures . . . 6

2 Simulating Kappa-models . . . 7
2.1 An overview of the stochastic simulation method . . . 7
2.2 Time advance with null events . . . 8

3 Counting complexes in acyclic contact maps . . . 10

4 Random Acyclic Graphs . . . 12

5 The effect of size constraints on the number of possible complexes . . . 13

6 The "stable rings" scenario . . . 15
6.1 The thermodynamic rationale for the "stable rings" scenario . . . 15
6.2 The implementation of the "stable rings" scenario . . . 16

7 The "no rings" scenario **18**

7.1 The implementation of the"no rings" scenario . . . 18

7.2 The relationship between "stable rings" and the "no rings" scenario . . . 20

8 Additional Results **20**

8.1 Alternative definitions of distance . . . 20

8.2 Structure-based affinities . . . 24

8.3 Results for SR simulations . . . 27

8.4 Results for different affinity scenarios . . . 27

8.5 Results based on adding "cryptic" cycles . . . 30

9 Comparison with Affinity Purification / Mass Spectrometry data **32**

References **34**

1 A Rule-Based Modeling Framework

1.1 Kappa

Rule-based representations [1–5] are promising for modeling systems based on compositional interactions that can give rise to astronomic numbers of possible molecular species [6]. To model the cytoplasmic SIN (cSIN), we employ a formal language called "Kappa" [2, 4, 7]. Although a formal definition of the language may be found elsewhere (for example, [7, 8]), we include a brief informal description for the sake of a self-contained presentation.

Kappa is a language designed to express interactions between protein agents in terms of rules that refer to partially decontextualized domains or "sites", much like rules of organic chemical reactions refer to partially decontextualized functional groups. Most of what we describe next also holds for other rule-based approaches, such as BNGL [1].

Agents and complexes. Agents are the atoms of the language and represent not full protein complexes but their individual protein constituents. Specification of an agent requires a name and a set of labeled sites—the interface of the agent. A site can have internal states that might represent post-translational modifications. In addition, a site may be bound to at most one site of another agent to form a complex, as sketched in Figure 1. Sites are best understood as representing resources for interactions (which are specified by rules, as explained below). Kappa aims at representing actions and their dependencies on state, without directly representing the structural underpinnings that make such actions physically possible. In other words, Kappa is meant to represent high-level mechanistic knowledge in a manner that enables the study of *process*, i.e. suites of events whose occurrence enables further events.

A complex is a Kappa expression in which the states of the constituent protein agents need not be fully specified, while a *molecular species* is a complex in which each agent occurs with its full

complement of sites in definite states. In other words, a Kappa expression is a pattern representing the set of species that match it; the former is an *intensional* object, the latter is an *extensional* one. The combinatorial explosion is tamed by replacing extensional lists by intensional expressions.

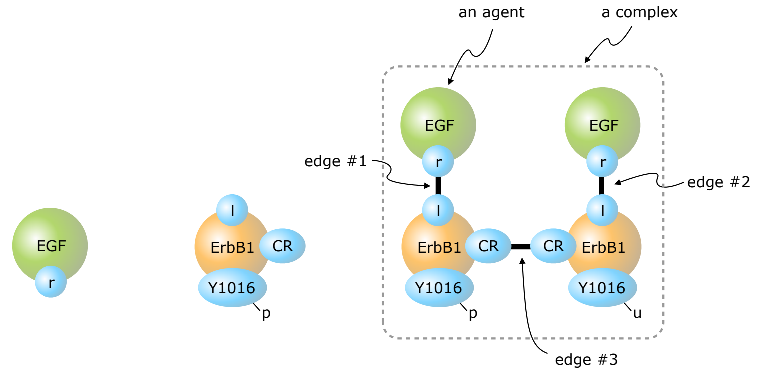

$$\mathtt{EGF(r), ErbB1(l, CR, Y1016_p), EGF(r^1), ErbB1(l^1, CR^3, Y1016_p), EGF(r^2), ErbB1(l^2, CR^3, Y1016_u)}$$

Figure 1. Kappa expressions. Bottom: The textual representation of a small reaction mixture containing 6 agents that make up three complexes, identified graphically above. The two complexes on the left are simple agents, while the complex on the right consists of 4 agents connected by pairs of identical superscripts at the corresponding binding sites. Top: An equivalent graphical representation. Agents and sites (blue) are labelled with names. An internal state, such as the phosphorylated state p of site Y1016 at agent ErbB1, is indicated by a labeled barb attached to a site. A mixture of molecular agents is a "site graph", i.e. a graph whose nodes are sets of sites (each set representing a particular agent) and whose edges are anchored by sites. A complex is a connected site graph, in which a site can bear at most one edge. The graphs do not represent geometric properties; they only convey connectivity and state information.

The meaning of "agent-based". The term "agent-based" has multiple meanings. One meaning refers to any representation of a system based on discrete entities (particles), typically for the purpose of stochastic simulation. Our use of the concept is more nuanced, as it refers to a level of structural granularity, not just discreteness. An analogy to chemistry might help. If molecules are considered to be agents, then the representation of molecular systems requires declaring an infinite alphabet—one symbol (a proper name) per molecular species. In contrast, if atoms are agents, then a very small alphabet and a few rules of *grammar* suffice to build (and thus compositionally name) an infinity of molecules. In both cases we deal with discrete entities and their interactions, but only the latter hinges on a structured language.

Rules. Sites represent capabilities for interaction, like binding and post-translational modification, specified by rules. The idea of a rule is to stipulate only the context required for an interaction (Figure 3 of the main text), along with rate information. Instead of directly writing reactions between exhaustively specified molecular species, we write rules that mention names of protein-agents and some, but not necessarily all, of their respective sites. In this way, a rule need only make explicit those aspects that are relevant to the interaction being described. More

specifically, the left hand side (LHS) of a rule is a pattern. The right hand side (RHS) defines the changes that occur when the LHS is matched in a mixture of agents. The difference between RHS and LHS is called the action of the rule. Sites mentioned on the LHS are said to be tested by the rule. Sites that are tested but not modified constitute the context of a rule's action. Because rules typically do not mention all the sites and states of an agent, they keep combinatorial complexity implicit. Yet, all possibilities are still realizable at the level of agents in the mixture to which the rules apply (see below). For example, if a substrate has four independent phosphorylatable sites, only one rule is needed to express the modification of any given site, whereas 8 reactions are necessary to express that same action in all possible contexts occasioned by the remaining three sites. Rules can be modified, refined or coarsened as needed to express new knowledge and new hypotheses.

Events. Rules are applied to a *mixture*, which is a large Kappa expression representing the contents of a reaction system at a particular time, as illustrated in Figure 2. The lens over the LHS pattern is meant to suggest that applying a rule requires searching for a match, which then creates a reaction instance between the matching complexes (Figure 2B). To apply a rule then means updating the states of the agents so identified in the mixture. Rules may modify several agents at once by invoking any of five elementary actions defined in Kappa: binding, unbinding, internal state modification, creation of an agent, and removal of an agent. In our specific study of complex formation, only binding and unbinding actions occur.

Models. A Kappa-model is a collection of rules and an initial mixture on which the rules act. At any given time, different rules may apply in distinct ways involving distinct reactant combinations. The dynamics generated by a Kappa model is therefore stochastic. A rule fires with a probability computed from the number of its matchings in the mixture (mass action) and its rate constant (section 2.1). The overall procedure for generating trajectories that are probabilistically compatible with the underlying master equation of stochastic chemical kinetics follows the Doob-Gillespie algorithm [9, 10] appropriately generalized to rules [11, 12].

Textual notation. The graphical rendering of Kappa expressions is equivalent to a textual notation, Figure 1. Although the graphical depiction appeals to instant visual comprehension, it is less convenient for in-text use.

Implementation and availability. The Kappa framework is an evolving suite of tools designed for navigating and exploring the structure and dynamics of complex signaling systems. It includes a graphical user interface, a scalable stochastic simulator, a set of procedures for analyzing the causal relationships between rules, a sampler of causal flows (pathways), and an exact coarse-graining procedure for converting a rule-based model into a corresponding set of differential equations whose variables refer to *sets* of molecular species that are distinguishable by the system. The framework and its source codes are available at www.kappalanguage.org.

1.2 Site graphs, contact maps, complexes, and molecular species

Site graph. A site graph is a graph in which nodes are *sets* of sites with edges connecting sites, Figure 1 of the main text. We can think of a site graph as adding additional structure on top of a standard graph by partitioning its nodes into sets. These sets then become the nodes of the site

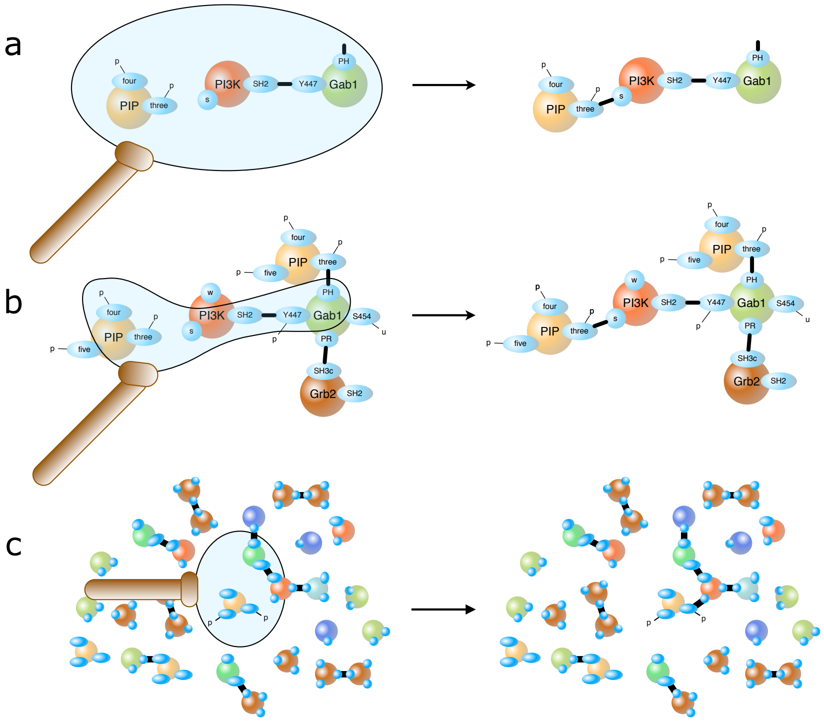

Figure 2. Rules, instances, and events in Kappa. A: A (fictitious) Kappa-rule specifying conditions for a binding event between two proteins PI3K and PIP. The lens symbolizes the pattern that must be matched for the rule to fire. **B:** A particular combination of complexes is seen to match the LHS of the rule in panel A, yielding a reaction instance. **C:** An event is a particular occurrence of a reaction instance, involving specific molecules in a mixture. Many matchings may be possible for any given rule in a mixture, and many different rules may be applicable at a given moment.

graph and the former nodes—now called sites—remain the endpoints of edges.

Contact map. A contact map is a site graph that summarizes statically the possible interactions given by the rules of a Kappa model. The nodes of the contact map are the agents (proteins) that occur in the model. An edge is placed between sites of two agents if the model contains a binding rule whose LHS can be satisfied given an initial condition and the other rules of the model. (The contact map has therefore a semantic content that requires, in general, a reachability computation [13].) Since a contact map summarizes possibilities, each agent type occurs exactly once, but a site may have more than one incident edge. The cSIN in Figure 2 of the main text is a contact map.

Complexes and molecular species. A complex of agents is a site graph (e.g. Figure 1) that is realizable given a contact map. A complex is intended to be a partial description of a molecular species that is compatible with the contact map of the model. As such, a complex may contain more than one occurrence of a given agent type, but every site can have at most one bond. A

molecular species is a complex that mentions the full complement of sites (and their states) for each agent.

Cycles. Cycles in the contact map have different meaning depending on whether the cyclical path of bonds touches the same site twice. If it does, the cycle in the contact map cannot give rise to a cyclical realization of it (i.e. a complex that is a ring), because in a complex a site can be used at most once. A "proper cycle" in the contact map is a cyclical path of bonds, starting and ending at the same node, which does not touch the same site twice. Such a cycle can give rise to infinite realizations of different complexes: each time the addition of a bond comes full circle in the contact map, the bond need not be to an agent already in the complex, but could be to a new copy, thereby creating polymers of any length.

1.3 Locality of rules and cyclical structures

Kappa rules are *local* in the sense that their context is self-contained: verifying that a combination of molecules in the mixture satisfies the LHS of the rule never requires information outside of the LHS. This has subtle consequences.

Consider the rule in Figure 3A of the main text. This rule, call it R, stipulates a binding action between an agent of type `A` with a free site `s` (in any internal state) and an agent of type `B` with a free site `p` (in any internal state). Agents `A` and `B` may have other sites, but their states do not figure in the context relevant to R. The fact that `A` and `B` are disconnected on the rule's LHS does not imply that their embeddings (matchings) into the mixture must end up disconnected, as shown in Figure 3B of the main text. Indeed, a condition that requires `A` and `B` to belong to different molecules is *inherently non-local*: Given two matching agents of type `A` and `B` in the mixture, a verification of the condition would require a scan of all (bond) paths originating at the matching agents to confirm that none of them ends up connecting `A` and `B` into a single molecule. This task has a worst-case computational cost bounded by the size of the mixture, not the LHS pattern of the rule. In the same vein, expressing in a local fashion that two agents are connected, requires specifying at least one connecting path. Without providing such a path, the condition that "`A` and `B` belong to the same molecule" is non-local, since identifying a connecting path requires a worst-case scenario of scanning of the whole mixture. Thus, "local" means that the size of the effective context of any action does not scale with the size of the system.

On the practical side, locality enables the development of efficient scalable simulators [11] and of static analysis tools [4, 7, 13] that greatly facilitate the process of modeling while also providing insight into possible behaviors of a rule collection. On the conceptual side, locality has a strong physical appeal, as the intuition behind the notion of "mechanism" is that of an action that depends on locally accessible information. While Kappa captures a meaningful level of process and attendant abstract locality, it is too simple to capture aspects of *physical* locality that must constrain our simulations of the cSIN, even though the cSIN data contain no information about the particular geometry of a putative complex.

As illustrated in Figure 3 of the main text, one consequence of locality in Kappa is a potential mismatch between the arity of a rule (the number of connected components on the LHS of a rule) and the actual molecularity of the reactions that the rule induces in the mixture. The mismatch

is potential, because whether it actually occurs depends on the initial mixture and the molecular species derivable from it by repeated rule applications. Binary Kappa rules are therefore annotated with two stochastic rate constants: a first-order, volume-independent rate constant and a second-order rate constant with a reciprocal dependence on the reaction volume. In the case of the cSIN, we automatically avoid arity mismatches as a by-product of addressing another consequence of locality: polymerization.

Whenever a binary rule can induce both uni- and bimolecular reactions (Figure 3B of the main text), the unimolecular scenario leads to the closure of cycles, whereas the bimolecular scenario can lead to the formation of polymers. In Figure 3B, the second `B`-agent that has been picked up in reaction 1 can subsequently bind to another `C`, which can bind a further `A`, and so on. Whenever cyclical structures can form in a local rule set, polymers can form too. Without proper constraints, mass action would end up favoring polymerization over ring closure. As can be seen from the contact map of the cSIN, Figure 2 of the main text, the potential for cyclical complexes is large and polymerization would be therefore rampant.

2 Simulating Kappa-models

2.1 An overview of the stochastic simulation method

The stochastic simulator developed for Kappa follows the logic of the well-known Doob-Gillespie procedure [9, 10], but is implemented in a manner that makes the core loop of the procedure scalable, i.e. independent of the size of the system and the number of possible molecular species that are implied by the rules, as detailed in [11].

In the following, recall that molecular species (complexes) are not represented as opaque units, but rather in terms of their constitutent protein agents. Thus, as far as the cSIN is concerned, our virtual cytoplasm consists of about three million protein agents (see main text), explicitly represented in computer memory one-by-one. The state of the cytoplasm at any particular time is a graph over this large set of protein agents, with individual complexes being subgraphs whose link structure is continually modified as the simulation proceeds in accordance with the rules of the cSIN and the NR and SR constraints. (The NR and SR constraints are detailed in sections 7 and 6, respectively, of this document.) At no point is there a counter keeping track of how many instances of a given complex the system contains. Such an analysis (which involves running a graph isomorphism to establish whether two complexes are the same) is only performed at specific time points when the simulator reports on the contents of the mixture. The memory usage of the simulator is thus bounded by the number of individual agents present, a number which generally remains roughly constant even if the simulation samples a large set of unique complexes.

We briefly sketch the basic approach to computing firing probabilities for rules. The Gillespie procedure is usually applied to interaction networks based on molecular species and the reactions that relate them. In Kappa, however, the conceptual units are patterns along with the rules that relate them. Recall that the agent `A` on the LHS of rule R in Figure 11 of the main text is (usually) a pattern, not a molecular species, since `A` might possess many sites other than `s`. The rule simply states that the binding between `A` and `B` occurs independently of whatever else `A` and `B` are bound to. This is precisely the empirical content of the cSIN.

The activity α_i of a *reaction* i is a mass action term that is a function of the number of instances in the system of each molecular species appearing on the LHS of reaction i. In the case of a *rule* i, we need to count the number of occurrences of the LHS pattern in the large graph that represents the mixture. This amounts to counting the many ways the (usually small) LHS graph of rule i can be embedded (matched) in the (usually large) mixture graph at time t. Let this number be $\theta_i(t)$. For example, the rule R in Figure 11 of the main text will have $n_{A(s)}n_{B(p)}$ embeddings in a mixture that contains $n_{A(s)}$ agents of type `A` whose site `s` is free and $n_{B(p)}$ agents of type `B` whose site `p` is free. Given a stochastic rate constant γ_i, the activity α_i of rule i is

$$\alpha_i(t) = \gamma_i \theta_i(t). \tag{1}$$

At the beginning of the simulation we incur an upfront cost by computing all matchings for all rules in the system, which are stored as pointers in appropriate data structures. Once the initial activity of each rule has been calculated, we proceed in the standard fashion:

1. Select a rule r with probability α_r/λ, where $\lambda = \sum_r \alpha_r$ is the total system activity.
2. Draw a time advance δt exponentially distributed with parameter λ, $p(\delta t) = \lambda \exp(-\lambda \delta t)$, and advance the simulated wall-clock time t to $t + \delta t$.
3. Select at random one matching of the rule r chosen in step (1).
4. Execute the action represented by the rule r selected in step (1).
5. Update the data structure of current matchings to reflect the loss and gain of matchings from the executed action.
6. Update affected rule activities and recompute the overall system activity λ. This is done very efficiently, taking into account causal relationships between rules [11].
7. Repeat.

2.2 Time advance with null events

A null event is an attempted rule application that is rejected on the basis of constraints that refer to aspects of an interaction that can only be known at run-time when the rule instantiates as a reaction involving a particular combination of reactants. The point of the Doob-Gillespie method is to avoid null events alltogether, since the choice in step (1) of the core loop (section 2.1) is intended to be among reactive events. Various constraints applied at runtime (such as the NR and SR constraints) may reject attempted reactions on the basis of properties that the products would have. Null events always incur a computational cost logarithmic in the number of choices in step (1) of section 2.1 above. But the question is how to advance simulated time when a null event has occurred.

The upshot is that when a null event occurs, simulated time must advance exactly as if the event had been reactive even though no change occurs in the system. This approach has been proposed

by Yang et al. [12], but without offering a proof of correctness, which we shall supply here for the sake of thoroughness.

A rule i is a source of null events if some attempts of applying it will be rejected. The activity of a rule i at time t is defined in terms of the set of potential next events—the occurrences of the rule's LHS in the mixture at time t, $\theta_i(t)$, see equation 1. We do not know which potential next events would be rejected by the constraint, as knowing them in advance would require computing the constraint for all of them, which is computationally much too expensive—in particular when rejection is rare. Rather, we work with the apparent activity of rule i, α'_i, which is always equal or greater than the true activity α_i based on potential next events that would be productive: $\alpha_i = \alpha'_i - \epsilon_i \alpha'_i$, with $0 \leq \epsilon_i \leq 1$ being the fraction, or likelihood, of potential null events among the potential next events induced by rule i:

$$\epsilon_i \equiv \frac{\alpha'_i - \alpha_i}{\alpha'_i}. \tag{2}$$

Let also $\lambda' \equiv \sum_r \alpha'_r$ be the total apparent activity of the mixture at time t.

First, if we choose the next rule i with probability α'_i/λ', then the probability that rule i is the next *productive* rule chosen is α_i/λ. This can be seen by considering that, if rule i is chosen, the probability of choosing a productive copy of that rule is α_i/α'_i $(= 1 - \epsilon_i)$. The probability that rule i is chosen and productive is thus $(\alpha'_i/\lambda')(\alpha_i/\alpha'_i) = \alpha_i/\lambda'$. The probability that the next productive rule is rule i is just the probability of choosing a productive copy of rule i divided by the total probability of choosing a productive rule. Since the total probability of choosing a productive rule is $\sum_i \alpha_i/\lambda' = \lambda/\lambda'$, we obtain $(\alpha_i/\lambda')(\lambda'/\lambda) = \alpha_i/\lambda$. Note that the probability of choosing the next productive reaction to be an application of any rule i is thus equivalent to the case one would obtain in the absence of clashes.

Next we must show that the distribution $p(\Delta t)$ of waiting times Δt between *productive* events, $p(\Delta t) = \lambda \exp(-\lambda \Delta t)$, results from using the distribution $p(\delta t)$ of waiting times between any events (productive and null) using the apparent total activity λ', $p(\delta t) = \lambda' \exp(-\lambda' \delta t)$.

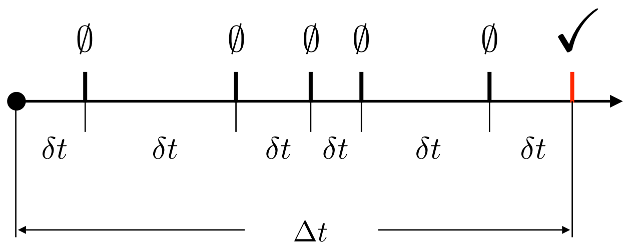

Figure 3. Null events. The time line of a sequence of five attempted events, all rejected on the basis of failing some test, and a sitxh event that is reactive (i.e. it alters the state of the system). Each increment δt (including the last one leading up the reactive event) is independently and identically distributed with an exponential probability density $p(\delta t) = \lambda' \exp(-\lambda' \delta t)$, as the parameter λ' is unaffected by a "non-event". The probability density of the sum of all these increments is therefore a Gamma distribution.

The distribution of waiting times until the next productive event can be written as

$$p(\Delta t) = \sum_{n=0}^{\infty} p(n)\Gamma(\Delta t, n+1, \lambda'), \tag{3}$$

where n sums over the number of null events that might occur before a productive event is chosen; $p(n)$ is the probability of choosing a sequence of n null events terminated by a productive event. Each of these events generates a time advance δt (Figure 3) based on the same exponential density $p(\delta t) = \lambda' \exp(-\lambda' \delta t)$, since the total system activity λ' remains unchanged throughout the null series up and including the productive event (after which λ' may change). The probability density for the sum of $n+1$ independent exponentially distributed random variables is a Gamma distribution. In our specific case:

$$\Gamma(\Delta t, n+1, \lambda') = \lambda' \frac{(\lambda' t)^n}{n!} e^{-\lambda' \Delta t}. \tag{4}$$

The probability of getting a series of n null events followed by a productive event is given by $p(n) = \epsilon^n (1-\epsilon)$, where $\epsilon \equiv \dfrac{\lambda' - \lambda}{\lambda'}$. Putting all of this together, we can rewrite equation 3 as:

$$p(\Delta t) = \sum_{n=0}^{\infty} \epsilon^n (1-\epsilon)\lambda' \frac{(\lambda' \Delta t)^n}{n!} e^{-\lambda' \Delta t} = (1-\epsilon)\lambda' e^{-\lambda' \Delta t} \sum_{n=0}^{\infty} \frac{(\epsilon \lambda' \Delta t)^n}{n!}. \tag{5}$$

The sum on the RHS of equation 5 is just the expansion for the exponential. This gives

$$p(\Delta t) = (1-\epsilon)\lambda' e^{-\lambda' \Delta t} \left(e^{\epsilon \lambda' \Delta t} \right) = (1-\epsilon)\lambda' e^{-(1-\epsilon)\lambda' \delta t} = \lambda e^{-\lambda \Delta t}. \tag{6}$$

We thus have that the probability of choosing a productive rule i is α_i/λ and that the waiting time distribution $p(\Delta t) = \lambda e^{-\lambda \Delta t}$, demonstrating that the approach to null events taken here results in a case equivalent to the Gillespie-Doob approach for the set of productive reactions.

3 Counting complexes in acyclic contact maps

To count the possible species that can be realized from a collection of binding rules with an *acyclic* contact map, we recast the contact map in terms of "views" that are local to each agent. (For a more general analysis and application of the local view concept, see [13].) In essence, creating local views amounts to prying each agent out of the contact map while retaining "bond stubs" (half edges) pointing to the agent and the site at the other end of the bond. Thus, if agent $\mathtt{A}$ connects at site $\mathtt{a}_1$ to agent $\mathtt{B}$ at site $\mathtt{b}_1$ and at site $\mathtt{a}_2$ to agent $\mathtt{C}$ at site $\mathtt{c}_1$, we can define a "fragment" $\mathtt{A}(\mathtt{a}_1^{\mathtt{b}_1@\mathtt{B}}, \mathtt{a}_2^{\mathtt{c}_1@\mathtt{C}})$ that contains two bond stubs representing surfaces complementary to fragments $\mathtt{B}(\mathtt{b}_1^{\mathtt{a}_1@\mathtt{A}}, \ldots)$ and $\mathtt{C}(\mathtt{c}_1^{\mathtt{a}_2@\mathtt{A}}, \ldots)$. For each agent we also generate all versions in which any number of sites lacks a stub, meaning that they will remain unbound. Two complementary surfaces can be plugged together, creating a larger fragment in which these surfaces are unavailable for further interaction. Surfaces that have not been paired with their complement are termed "open", otherwise they are "closed". The set of open surfaces constitutes the interface of

a fragment. These definitions extend to complexes.

Our counting procedure, specified below, combines fragments $\mathcal{F}$ into bigger fragments (with new interfaces), starting with the initially provided local views. At every iteration there is a "current set" of fragments that constitutes the material for further combinations in the next iteration. Yet, rather than thinking in terms of fragments, we shall think in terms of interfaces. At the start of the process, we collect all fragments (which at that point are local views) with the same interface into a set and determine its size. This results in a collection of interfaces $\mathcal{I}$, each associated with a cardinality $|\mathcal{I}|$ reporting the number of fragments with that interface. Instead of combining fragments, we combine interfaces and update the associated cardinalities, thus keeping track of the number of fragments with that interface generated up to the current iteration.

The trick is to exploit the fact that any complex is, by assumption, an acyclic complex, which is to say a tree structure. To systematically build a tree, we grow it by repeatedly combining two interfaces, one of which we require to have *exactly one surface*. This can always be done, since an acyclic graph has at least one node with at most one edge. Hence we start with the terminal nodes of the graph (which are connected by exactly one edge or surface). If the other node, call it `Y`, has two surfaces, then this combination will use up one, leaving a fragment with only one surface that can be used to further grow the graph in the next step. If `Y` has multiple surfaces, we build the acyclic subgraphs connecting to each one of these surfaces in exactly the same manner, starting from their terminals. Once each subgraph is completed it has exactly one available surface to connect with `Y`. When all but one surfaces of `Y` are occupied, our tree has grown to include `Y` and has one surface left to be combined with another acyclic subgraph. When all surfaces of a growing tree are exhausted, we have a complex. Since we are not combining fragments but interfaces, we are effectively operating with sets (whose members, however, are never explictly represented!), enabling us to easily keep track of the cardinality of the interface resulting upon combination. (Do not confuse the cardinality of an interface, i.e. the number of implicitly generated fragments that possess that interface, with the size of an interface, i.e. the number of its surfaces.) Keeping these preliminaries in mind, our procedure reads as follows.

1. From the initial set of local views, construct a list of interfaces, each of which is associated with a cardinality reporting how many local views (fragments) exist with that interface.

2. Loop over all interfaces $\mathcal{I}$ *that have exactly one surface*. If none are present, go to step 4. If a fragment with interface $\mathcal{I}$ is to appear in a complex, it must interlock with some fragment with interface $\mathcal{J}$ containing a surface complementary to $\mathcal{I}$'s. Thus, we combine $\mathcal{I}$ with each interface $\mathcal{J}$ that has a complementary surface, generating an interface $\mathcal{K}$.

   The interface $\mathcal{I}$ is discarded (but not $\mathcal{J}$) and the interface $\mathcal{K}$ is added to the current set. The cardinality associated with $\mathcal{K}$ is updated to $|\mathcal{I}| \cdot |\mathcal{J}| + |\mathcal{K}|$, where $|\mathcal{K}|$ is the prior cardinality of $\mathcal{K}$, if $\mathcal{K}$ already existed in the prior set of interfaces.

3. Repeat step 2.

4. The current set contains an empty interface ε, and its cardinality is the number of possible complexes that can be formed with the local views obtained from the initial contact map.

By using arbitrary-precision arithmetic, we can *exactly* determine the astronomically large numbers of complexes that arise from the artificial contact maps generated in the next section.

We have omitted one final issue: symmetries (automorphisms). The only symmetry that can arise in an acyclic complex stems from dimerization, that is, the binding of two copies of the same agent type at sites with the same label, as in the dimerization of many receptors in signaling cascades. We refer to this as "self-binding", because it shows up as a loop in the contact map. (Notice that a loop returning to the same site at which it originated is not a cycle, as it cannot be realized as a cyclical complex; rather, such a loop results in a dimer.) This case is easily taken care of by redefining "interfaces of size 1" in step 2 of our procedure as interfaces that have one non-self-binding surface and possibly one self-binding surface. The combinations on the non-self-binding surface proceed as described above. In addition, we need to account for combinations on the self-binding surface, and these contribute $|\mathcal{I}| \cdot (|\mathcal{I}| - 1)/2$ to the cardinality of the resulting interface.

4 Random Acyclic Graphs

We generate Random Acyclic Graphs (RAGs) using the following iterative procedure. One begins at step 1 with a pair of nodes (named $\mathtt{A_0}$ and $\mathtt{A_1}$), each with a single site labeled $\mathtt{A_0.s_1}$ and $\mathtt{A_1.s_1}$, respectively, connected by an edge (representing a possible binding action). At each subsequent step n, a new node $\mathtt{A_n}$ is added to the graph. $\mathtt{A_n}$ is created with a single site, $\mathtt{A_n.s_n}$. A node $\mathtt{A_i}$ is then chosen at random with equal probability from the set of existing nodes $\{\mathtt{A_0}, \ldots, \mathtt{A_{n-1}}\}$, and a new site is created on $\mathtt{A_i}$, labelled $\mathtt{A_i.s_n}$. Finally, an edge is created between $\mathtt{A_i.s_n}$ and $\mathtt{A_n.s_n}$. By construction, this procedure results in an acyclic site graph. A so-generated graph with N nodes has $N-1$ edges, which corresponds to a fairly low edge density $\rho = 2N^{-1}$. Indeed, with $N = 167$ nodes $\rho \approx 0.012$, which is considerably smaller than the edge density observed for the cSIN ($\rho \approx 0.039$).

To provide a more accurate comparison to the cSIN, we extend the previous procedure by a second phase in which more edges are added. In phase 1, a graph is generated with N nodes, exactly as previously described. In phase 2, new edges are added to this graph in a manner that avoids proper cycles (recall that a proper cycle in a site graph is a path that starts and ends at the same node and consists of bonds that do not touch the same site twice, section "Methods"→"Dealing with network cycles" in the main text). To set up phase 2, define $\mathcal{S}_n$ to be the set of all sites that existed prior to step n in phase 1, $\mathcal{S}_n = \{\mathtt{A_j.s_k} \mid 0 \leq j < n, 1 \leq k < n\}$ (with the proviso that $\mathtt{A_j.s_k}$ exists, since the sites of $\mathtt{A_j}$ are not labelled consecutively). Also, define $\mathcal{S}_{\mathtt{A_i}}$ as the set of sites belonging to a particular node $\mathtt{A_i}$. To add new edges, we first choose a node $\mathtt{A_i}$ at random (uniformly) from the set $\{\mathtt{A_2}, \ldots, \mathtt{A_N}\}$. We then choose another node $\mathtt{A_j}$ from the set $\{\mathtt{A_2}, \ldots, \mathtt{A_{i-1}}\}$ (i.e. a node *older* than $\mathtt{A_i}$). Finally, we choose a site $\mathtt{s_k}$ at random with uniform probability from the set $\mathcal{S}_i \cap \mathcal{S}_{\mathtt{A_j}}$, that is, a site $\mathtt{A_j.s_k}$ from the sites on $\mathtt{A_j}$ that existed prior to step i. An edge is then placed between $\mathtt{A_i.s_i}$ (the only site on $\mathtt{A_i}$ that exists at step i of phase 1) and $\mathtt{A_j.s_k}$; if this edge already exists, another node is chosen at random from the set $\{\mathtt{A_2}, \ldots, \mathtt{A_{i-1}}\}$, until a new edge is successfully placed or all possibilities in $\mathcal{S}_i$ have been exhausted; at that point a different $\mathtt{A_i}$ is chosen at random. These steps are repeated until the edge density of the RAG is approximately equal to the desired edge density of the cSIN, $\rho \approx 0.039$.

This way of adding edges cannot produce contact maps with proper cycles. Any newly added edge in phase 2 is always between a site $\texttt{A}_\texttt{i}.\texttt{s}_\texttt{i}$ ($2 \leq i \leq N$) and a node $\texttt{A}_\texttt{j}$ with $j < i$ that belongs, by construction, to an acyclic subgraph generated before step i in phase 1. The only "return" from a node in that subgraph back to $\texttt{A}_\texttt{i}$ was created exactly at step i and involves the same site $\texttt{A}_\texttt{i}.\texttt{s}_\texttt{i}$, and is, therefore, not a proper cycle. A complex constructed from such a contact map can never be a ring, since in a complex (unlike in a contact map) any site can be bound at most once (in particular $\texttt{A}_\texttt{i}.\texttt{s}_\texttt{i}$), see definitions in section 1.2.

In practice we find that this procedure efficiently generates RAGs for $N \gtrsim 60$ at an edge density similar to that of the cSIN. We create sets of RAGs with varying number N of nodes but a fixed edge density $\rho \approx 0.039$. Each point in Figure 4B of the main text reports the avarage number of possible complexes as determined from 10 independently generated RAGs with a given N.

5 The effect of size constraints on the number of possible complexes

In the above analysis, the assumption is made that all possible complexes can be physically realized by the proteins and their interfaces represented in the (acyclic) cSIN-like contact maps. Although we include steric constraints at runtime (particularly in the "stable rings" scenario, section 6), the counting algorithm described in section 3 does not account for steric effects that might prevent the formation of certain complexes. In this section we perform a simple calculation to explore the consequences that steric constraints might have on the total number of molecular species that an interaction network could form. The case we consider here represents a fairly strong constraint, in which steric effects become more and more prominent as complexes get larger. Given that the surface area of a complex will tend to increase with increasing size, this might not represent the most realistic situation. We nonetheless consider this model to demonstrate that even strong steric constraints do not curtail combinatorial complexity significantly, unless the parameters of the model are set to extreme values.

One approach to assessing the reduction in the number of complexes from higher-order steric constraints would be to enumerate the set of possible complexes and then remove some fraction of these (as a function of complex size). This fraction would represent a parameter of the model. Such an analysis cannot be performed directly, because the large number of possible complexes prevents their explicit enumeration. However, based on the initial explicit enumeration depicted in Figure 4A of the main text, we argue that the total number of complexes scales (approximately) in an exponential fashion with complex size, leading us to posit:

$$N = \sum_{s=1}^{M} N(s) = \sum_{s=1}^{M} be^{as}, \tag{7}$$

where N is the total number of complexes that can be formed by the network, $N(s)$ is the number of complexes of size s with s ranging from 1 (monomers) to M, the size of the largest complex that the network could form. a and b are free parameters. Computing the geometric series in equation 7, we can write:

$$N = b\frac{e^{aM} - e^{a}}{e^{a} - 1}. \tag{8}$$

The model represented by equation 7 states that $N(1) = be^a$ and $N(s) = e^a N(s-1)$, expressing an exponential progression as one forms complexes of size $s+1$ from complexes of size s. Let us now assume that the addition of a protein in growing complexes from size s to $s+1$ encounters a steric hindrance that reduces the number of attainable complexes by a factor of p ($0 < p \leq 1$). This assumption means that $N(1) = be^a$ and $N(s) = pe^a N(s-1) = bp^s e^{as}$. Thus,

$$N = \sum_{s=1}^{M} bp^s e^{as} = b\frac{e^{(a+\log p)M} - e^{a+\log p}}{e^{a+\log p} - 1} \tag{9}$$

where we see that the effect of p is to reduce the effective value of a by $|\log p|$.

To provide a sense for how large this effect might be, we estimate values of b and a for the RAGs discussed in section 4. Taking a network with 167 nodes, we know that the number of monomers will be 167; this gives us $b = 167e^{-a}$. For RAGs without any steric constraints (i.e. with $p = 1$), we have that $N \approx 10^{40}$ (see Figure 2 of the main text). Assuming that $M \approx 40$ (see Figure 6 of the main text) allows us to solve equation 8 numerically for a. Using Mathematica [14], we get $a \approx 2.23$ and thus $b \approx 17.96$. Using these values, Figure 4 depicts how the total number of complexes will change as p is decreased (thus increasing the steric hindrance at every growth step).

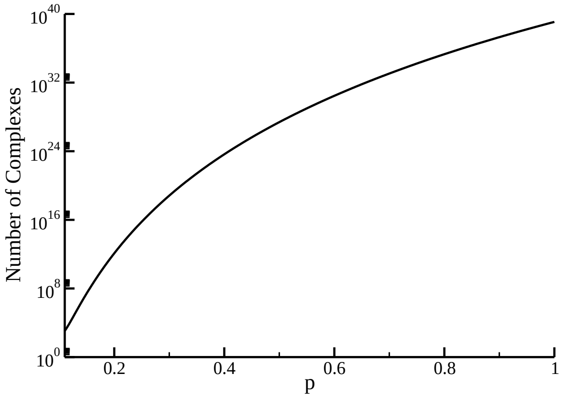

Figure 4. Effect of steric constraints. The plot shows the effects that higher-order geometric and steric constraints might have on the total number of possible complexes a network could form. In this case, we assume that the number of complexes increases exponentially with complex size up to some maximal complex size. p represents the fraction of complexes that cannot be formed due to steric constraints when attempting to bind a protein to complexes one size smaller. The black line is calculated using equation 9, with parameters $a = 2.23$, $b = 17.96$ and $M = 40$ chosen to approximate the case of a RAG with ~ 167 nodes. Note that a sizable fraction ($\sim 89\%$) of complexes must be sterically prevented *at each size* in order to produce numbers similar to the total number of unique complexes observed in a single simulation (i.e. 10^4, Figure 5A in the main text).

From Figure 4 we can see that even when p is fairly small the network is still likely to produce a very large number of possible complexes. Even if only 20% of complexes of a given size can be realized, the total number is still $\sim 10^{12}$. This approximate calculation suggests that steric constraints would have to be incredibly strong in order to reduce the number of molecular possibilities to numbers that would allow their simultaneous sampling by a cell.

6 The "stable rings" scenario

6.1 The thermodynamic rationale for the "stable rings" scenario

The SR scenario posits that all cycles in the contact map of the cSIN correspond to physical structures whose bonds along the cycle backbone can be satisfied simultaneously whenever a complex containing the elements of the cycle arises, regardless of cycle length. In order to implement the SR case, we must first calculate the rate at which cycles should close (i.e. the rate at which the intramolecular `A-B` bond missing on the left of Figure 11B in the main text should form). We follow the work of Saiz and Vilar [15] by defining the standard free energy change upon binding of two proteins (ΔG_b^0) as:

$$\Delta G_b^0 = \Delta G_p^0 + \Delta G_i^0, \tag{10}$$

where ΔG_p^0 represents the positional entropy loss entailed when taking two proteins that can freely diffuse around a particular molar volume and confining them to a given binary complex. ΔG_i^0 represents the free energy of the specific molecular interactions in the complex, including contributions from the desolvation of the two protein interfaces and the molecular contacts (e.g. electrostatic and Van der Waals interactions) formed upon binding. We assume that $\Delta G_b^0 < 0$ for every pair of interacting surfaces in the cSIN *independently*; that is, for every edge in the graph we assume that the implied binding reaction will favor the bound form even when no other proteins from the cSIN are included in the system. As discussed in "Methods" (main text), we employ various interaction affinities, but here we focus on a case in which every reaction has a dissociation constant of 10 nM. If we assume the reactions are taking place at an absolute temperature of ~ 300 K, and that the standard molar positional entropy loss upon binding is ~ 9 kcal mol^{-1} [16], a 10 nM dissociation constant implies that $\Delta G_i^0 \sim -21$ kcal mol^{-1}.

The above calculation is based on the assumption that two proteins are binding to one another in a bimolecular fashion. Cycle closure, however, is a unimolecular reaction. Moreover, in the SR case, we assume that complexes can structurally satisfy all of the interactions in the cycle simultaneously. This implies that the positional entropy loss of the cycle closure reaction is ~ 0; that is, all the members of a particular complex are already constrained by being bound to one another, such that the formation of the final bond does little to change the positional entropy of either protein that participates in it. The change in free energy represented by this unary reaction is thus $\Delta G_b^0 \sim \Delta G_i^0 \sim -21$ kcal mol^{-1}. This implies that such rings are very stable, as has been argued elsewhere [15, 17]. Indeed, if we designate the forward rate of cycle closure as u_+ and the cycle opening rate as u_-, then the free energy of cycle closure implies that $u_-/u_+ \sim 10^{-16}$.

If we assume that the cycle opening rate and the off rate k_- of the binary reaction are approximately equal, we have $u_-/k_+ = 10^{-8}$, so the binary binding rate k_+ must be much smaller

than the cycle closure rate: $u_+ \sim 10^8 k_+$. Our reasoning, so far, applies to deterministic rate constants. In a stochastic setting, bimolecular rate constants, such as k_+, are volume dependent [10]. Let κ_+ denote the stochastic binding rate constant expressed in units of molecule^{-1}s^{-1}. If the deterministic k_+ is in the usual units of $M^{-1}s^{-1}$, then $\kappa_+ = k_+/(N_A V)$ with N_A denoting Avogadro's number and V the reaction volume. In the present work, we take $V = 4.2\,10^{-14}$ L as the volume of a haploid yeast cell [18], which yields $u_+ \sim 10^{18}\kappa_+$.

We are led to conclude that cycle closure is so fast that, were it to be included explicitly in the simulation, most of the events at steady state would consist of cycle openings and closings. Moreover, once formed, cycles are so stable that they will tend to persist. Using the parameter values of the main text ("Results"→"Network Dynamics"), the time scale of the full excision of one member from its cycle ("cycle decay") is likely to be on the order of 10^{14} time units. Our longest simulations stretch over ~ 100 time units, meaning that on the time scale relevant to this study, a cyclic structure will not decay once it has formed. This is true not only for the 10 nM dissociation constant used in our illustrative calculation, but for every interaction affinity case discussed in the main text. We therefore implement cycle closure at an infinite rate as in the work of [17], i.e. an SR simulation is defined by $u_-/u_+ = 0$.

One intriguing consequence of the thermodynamics of cycles is the fact that a set of proteins can form a very stable ring even if one or more of the bonds in the cycle is fairly weak. This leads to a situation in which a high-throughput screen, such as Yeast-2-Hybrid, might miss interactions that are not stable on their own but are stable in the context of a proper cycle. We discuss this possibility further in section 8.5.

6.2 The implementation of the "stable rings" scenario

An implementation of the SR policy at the level of local Kappa rules would require an enumeration of all possible cycle closures. Even if such rules could be generated automatically, their number would be huge. We therefore implement the SR policy as a global directive to the simulator. Whenever a binding reaction occurs, the newly formed complex is explored to determine if it contains free sites that could bind any free sites on the protein that just entered the complex. If one or more such sites exist, they bind one another instantly without advancing simulated wall-clock time, which corresponds to a reaction with an infinite forward rate [10]. If a site (or sites) can participate in more than one cycle within the complex under consideration, the cycles that are actually formed are chosen at random with equal probability. Once a cycle is formed, any bonds in it are prevented from matching a dissociation rule, since such an event would immediately be reversed by an infinite-rate cycle closure event.

Setting the rate of cycle closure to infinity prevents many polymerization reactions from occurring, since complexes like the open triangle C_1 in Figure 5 immediately react along route 2 and never linger to pick up another `B` along route 1 to form C_2, which nucleates a polymer. Yet, the problem is not fully eliminated, as there is another route to C_2. A local binding interaction between the dimers $\texttt{A}(\texttt{p}, \texttt{s}^1), \texttt{B}(\texttt{p}^1, \texttt{s})$ and $\texttt{C}(\texttt{s}, \texttt{p}^1), \texttt{B}(\texttt{s}^1, \texttt{p})$ on the RHS of reaction 3 in Figure 5 also generates the troublesome C_2. This reaction must be prevented on the basis of a steric consistency argument. Recall that the assumption of the SR scenario states that whenever all members of a cycle are in the same complex their geometry satisfies all their bindings. Hence a

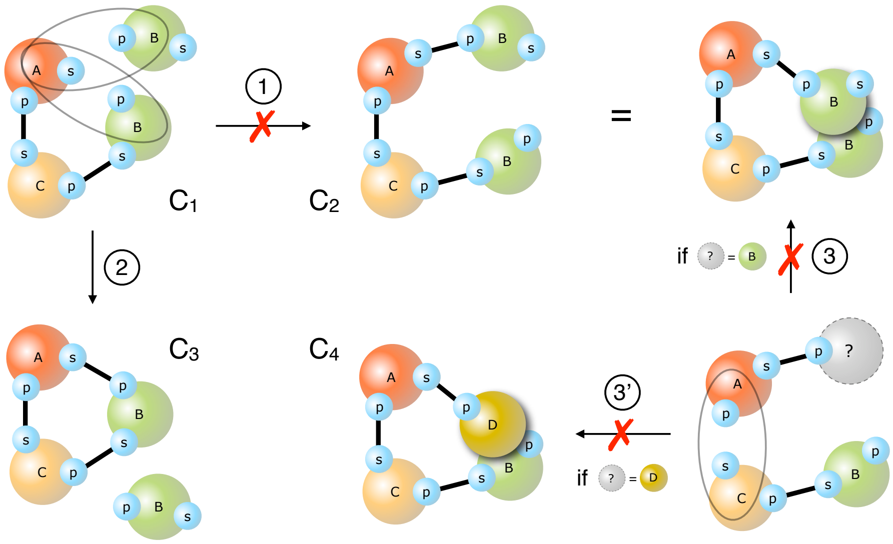

Figure 5. The excluded volume constraint. The left of the figure illustrates the infinite cycle closure condition. C_1 is a complex in which protein A can bind protein B. Because of the local nature of binding rules (corresponding to the local nature of the information in the SIN), there are two possibilities, here indicated by ovals: A binds the B that is already in the complex (unimolecular reaction 2), or A binds a B that is not in the complex (bimolecular reaction 1). Based on thermodynamic arguments (section 6.1), the SR constraint posits that whenever a complex contains members that could form a cycle (as we assume to be the case for protein agents A, B, and C), the cycle closure occurs immediately (reaction 2), thus preventing reaction 1. The undesirable complex C_2 is also an intermediate towards polymerization. (In the case of an ambiguity in the molecularity of a local binding interaction, it is always the case that the unimolecular reaction is a ring closure, whereas the bimolecular interaction may be a step towards polymerization, depending on the context.) However, the polymeric intermediate C_2 could also be formed by reaction 3, which involves the binding (indicated by the oval) of two dimers that would bring together into one complex all the members of a cycle, yet with one of the sites required for cycle closure (here s of A) already occupied. This is a steric contradiction that must be prevented, regardless of whether the outcome is a polymerization intermediate (as when A is bound to a B), reaction 3, or not (as when A is bound to a D), reaction 3'. Although not a polymerization intermediate, the latter product is prevented nontheless because of steric consistency. The SR scenario is therefore more stringent than the sole prevention of polymers.

dimerization as in reaction 3 of Figure 5 would be sterically inconsistent, as it brings together all members of a possible cycle (proteins A, B, and C) with one member (A) already being occupied at a binding site (s) that must, by assumption, be in steric juxtapostion with its binding partner in the potential cycle (here the B bound to C). Consistency, therefore, requires that the B bound to A (or the B bound to C) prevents reaction 3, as it would end up occupying the same volume already occupied by the other B. This is suggested in Figure 5 by a pictorial clash in the rendering of C_2 emerging from reaction 3, but the reader should not forget that these diagrams are graphs with no geometric content. The same argument holds, of course, if A were bound at site s to a protein

other than B, say D, as in reaction 3' of Figure 5. The offense to the SR assumption comes from the occupancy of any site (within an attempted complex) that *would have* bound at infinite rate another site in the complex to close a cycle. We refer to this constraint as the "excluded volume" test. It, too, is implemented as a directive to the simulator: Any attempted binding is inspected at run-time to determine whether the resulting complex contains sites that offend the SR assumption in the sense just described. Simulations denoted as "stable ring" scenarios in this supplement are performed using the set of binary local rules derived from the cSIN (e.g. rules as in equation 2 of the main text) together with two amendments applied at run-time: (i) cycles close infinitely fast and are forever stable, and (ii) attempted complexes must pass the excluded volume test. Rejections of attempted reactions based on (ii) are treated as null events, see section 2.2.

Using Figure 5 as a guide, the infinite closure of cycles means that as soon as B binds C to form C_1, C_2 occurs. C_1 never enters the mixture. Structurally, this means that the incoming B really forms two contacts at once (by our idealized assumption of perfect juxtaposition). However, based on the binding rules in the system, there are two independent ways for B to close the ring: by first binding to C (as on the left of Figure 5) followed by immediate ring closure or by first binding to A followed by immediate ring closure. The independence adds up to twice as fast a ring "discovery" kinetics than is warranted by our assumption that both bonds form at the same time. To recapture the correct kinetics, each time an agent binds a complex in an event that happens to create the opportunity for a ring closure, we reject the proposed binding of the agent with probability 1/2. In this way, we effectively divide the ring discovery rate by 2 without the need of knowing a priori which events are potential ring closures, which is combinatorially prohibitive. The price, again, is the creation of null events, see section 2.2.

A consequence of preventing polymerization in the SR scenario is the avoidance of any molecularity ambiguity of local binary rules. In the SR scenario, productive reactions induced by binary rules will always be bimolecular, and are therefore assigned a single bimolecular stochastic rate constant.

7 The "no rings" scenario

The rationale behind this scenario is to *be agnostic about the nature of steric constraints that prevent the formation of polymers.* We simply prevent polymers by fiat, using a non-local constraint illustrated in Figure 6.

7.1 The implementation of the"no rings" scenario

Polymerization requires the coming together of parts, each of which with at least one copy of the same protein as the other. But the reverse is not true: it is not the case that the binding of parts that share a protein type necessarily leads to polymerization. It depends on how the the copies of the common type are bound inside the complex. Figure 6 depicts a binary local rule representing the binding of A and B. Suppose the LHS pattern of the rule is matched (grey oval) in the mixture by a pair of complexes as shown on the lower left of Figure 6, both of which contain a copy of protein Z (a protein with at least two binding sites). The wiggly lines stand for an arbitrary intervening set of connected proteins in each complex. The complex resulting from the binding of

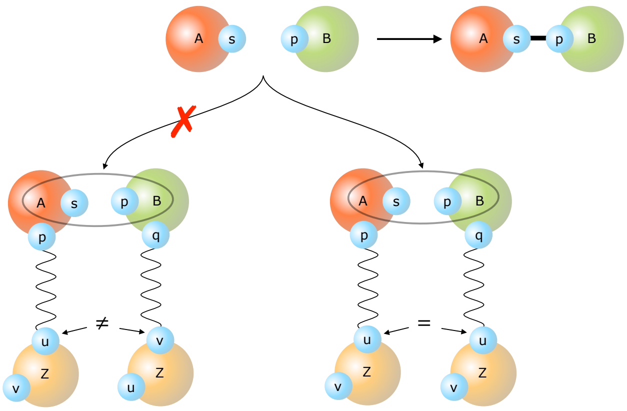

Figure 6. The no-polymerization constraint. The figure illustrates a syntactical criterion for rejecting binding interactions that would lead to the formation of polymers: The binding of complexes with duplicate parts (here `Z`) must be prevented whenever there is a path connecting them on *different* sites. See the text for a detailed explanation.

these two parts obviously contains two `Z`'s for which there is a connecting path – a path being a sequence of bonds representing an instruction for traveling through the complex from a particular site at a source protein to a particular site at a target protein. If a connecting path between the two copies of protein `Z` starts and ends at different sites of `Z`, the complex constitutes an intermediate in a polymerization process and must be prevented by rejecting the attempted binding. To see this, assume, as shown in Figure 6, that `Z` is connected by some path to `A` starting at `Z`'s site `u`, in pseudo-notation: `v.Z.u`∿∿∿∿`A`, while `u` is free on the copy of `Z` in the other part, `B`∿∿∿∿`v.Z.u`. Clearly, the new complex `v.Z.u`∿∿∿∿`A-B`∿∿∿∿`v.Z.u` could grow another copy of ∿∿∿∿`A` on `Z`'s free `u` site. The very existence of the `v.Z.u`∿∿∿∿`A` complex proves that this is possible given the set of local rules available to the system. Yet, if both copies of `Z` are tied up at the same site `u`, as shown on the lower right of Figure 6, they could not function as a platform for growing a polymer.

As a consequence of this constraint, complex C_4 in Figure 5 is allowed, but not C_2. However, an "isomer" of C_2, in which `A` is bound to `B` on its site `s` passes the no-polymerization test. The constraint depicted in Figure 6 prevents all polymerizing complexes and is more subtle than a simple ban of complexes that contain duplicates of proteins.

Rejected attempts at reaction based on the NR (or SR) constraint result in null events that are handled as detailed in section 2.2. In the NR and SR scenarios we have that binary binding rules always correspond to bimolecular reactions in the mixture, avoiding any potential mismatches in reaction arity (Figure 3 of the main text).

In sum, the NR scenario prevents polymerization by fiat, catching all higher-order geometric constraints that we cannot directly express in Kappa. However, neither the SR case nor the NR case is likely to represent the reality of complex formation in the cell. Some of the cycles in the contact map of the cSIN might represent SR complexes, others might follow the NR scenario or perhaps even give rise to polymers of limited size.

7.2 The relationship between "stable rings" and the "no rings" scenario

As noted in section 1.3, polymerization and ring closure are flip sides of the same coin. The trick in the SR scenario is to prevent polymers by invoking the steric and kinetic consequences that arise when proper cycles in the contact map are interpreted geometrically as rings. In the NR scenario, however, we prevent polymers purely logically—through the constraint depicted in Figure 6. Consider that constraint in the context of Figure 5. The no-polymerization criterion prevents the bimolecular reaction 1 between `A` and site `p` of `B` because there already is a path from `A` to a different site of another `B`. Note, however, that the same *path pattern* arises upon cycle closure, reaction 2, except that the path starting at `B` loops back to the same rather than a different instance of `B`. From a purely formal standpoint, if the constraint is to forbid certain paths, then, perhaps, cycle closure should be prevented as well, as it relies on the same forbidden path pattern. This "formal" argument actually blends with a structural argument. If the juxtaposition between `A` and `B` in C_1 (Figure 5) were perfect for ring closure, we would be back in the SR scenario. For the NR case to be different, we are forced to assume that the juxtaposition between `A` and `B` is *not* conducive to ring closure. This represents a case in which either the complex is very flexible, making ring closure entropically costly, or the `B`-binding site of `A` points in a different direction than the location of the resident `B`. The latter is compatible with the no-polymerization constraint, which allows `A` to indeed pick up an additional `B` (with the proviso on binding sites), see Figure 6 (where `Z` plays the role of `B`). Thus, in addition to preventing polymerization, the NR scenario also sets $u_+ = 0$, which is implemented by rejecting every binding reaction that would result in the closure of a cycle.

8 Additional Results

8.1 Alternative definitions of distance

In the main text, we define the "normalized distance" between two cells as:

$$d(i,j) = \frac{\mid C_i \,\Delta\, C_j \mid}{\mid C_i \cup C_j \mid} \tag{11}$$

where C_i is the set of unique complexes in cell i, $C_i \,\Delta\, C_j$ is the symmetric difference between C_i and C_j (i.e. $(C_i - C_j) \cup (C_j - C_i)$) and $C_i \cup C_j$ is the union of C_i and C_j. This represents a very natural distance since $d(i,j)$ is just the probability that a complex in either i or j will only be found in one of the two cells.

One could consider several alternative definitions of distance. For instance, if we think of the "complexome" of a cell as a string of 1's and 0's – with a 1 in a particular sequence position indicating that the presence of the corresponding complex, and a 0 indicating its absence. In this representation, $\mid C_i \,\Delta\, C_j \mid$ is just the "Hamming Distance" between the two complexome strings. It is natural to normalize the Hamming Distance by the distance we would obtain if the sequences were "orthogonal" in the complexes they contain (i.e. if every 1 in cell i corresponded to a 0 in cell j and vice versa), which yields:

$$h(i,j) = \frac{\mid C_i \,\Delta\, C_j \mid}{\mid C_i \mid + \mid C_j \mid}. \tag{12}$$

This definition is very similar to equation 11 and yields essentially the same general result (see Figure 7.)

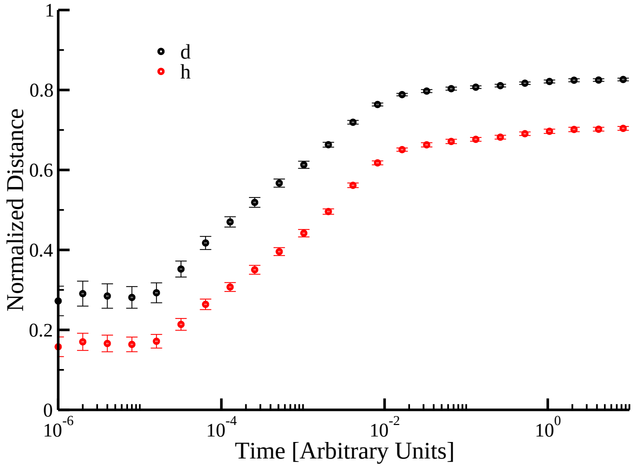

Figure 7. Comparing different definitions of the distance between cells. This figure is based on NR simulations with a uniform K_D of 10 nM. Each point represents an average over all unique comparisons between 15 independent simulations and the error bars correspond to $\approx 95\%$ confidence intervals. The black curve is calculated using equation 11; this data is equivalent to that displayed in Figure 5B of the main text. The red curve is calculated using equation 12. As one would expect, the two curves are very similar, with the normalized Hamming Distance giving distances somewhat smaller than the normalized distance discussed in the text. This derives from the fact that the normalizing factor in the Hamming Distance case is always greater than or equal to the normalization factor for $d(i, j)$; i.e. $\mid C_i \mid + \mid C_j \mid \geq \mid C_i \cup C_j \mid$.

Both of the above distances do not, however, weigh differences in the *copy number* of a particular complex. A complex that occurs in both cells i and j contributes the same weight to the overlap, regardless of the difference in the number of copies with which it occurs in each of these cells. We assess differences between cells that arise from copy number variations by considering two additional definitions of the distance. In the first case, we define a distance H_N which is similar to the Hamming distance but contains information about the differences in abundance of complexes:

$$H_N(i, j) = \sum_k \frac{\mid N_k(i) - N_k(j) \mid}{N_k(i) + N_k(j)} \tag{13}$$

where $N_k(x)$ indicates the copy number of a unique complex of type "k" in cell "x", $\mid N_k(i) - N_k(j) \mid$ is the absolute value of the difference in copy number for a complex of type k between cells i and j, and k ranges over all the types of unique complexes in cells i and j. Like the Hamming distance, equation 12, each term of the sum in equation 13 assumes a value between 0 and 1. However, two cells that both contain a particular type of complex k do not contribute to

the Hamming distance $h(i,j)$, equation 12, whereas such cases will increase the distance $H_N(i,j)$, equation 13, whenever $N_k(i) \neq N_k(j)$. As in the case of $h(i,j)$, two orthogonal cells will have a maximal distance of $H_N(i,j) = \mid C_i \mid + \mid C_j \mid$, which we use as a normalizing factor, yielding:

$$h_N(i,j) = \frac{H_N(i,j)}{\mid C_i \mid + \mid C_j \mid} \tag{14}$$

which is analogous to the normalized Hamming distance, equation 12. Equation 14 produces similar results to equation 12 (see Figure 8), with $h_N > h$ as one would expect.

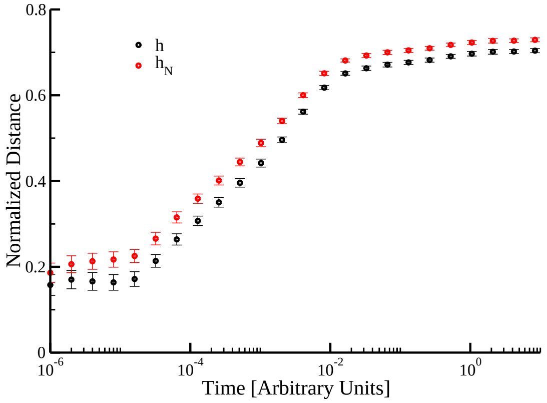

Figure 8. Comparing the Hamming distance with a similar distance definition that includes differences in copy number. This figure is based on NR simulations with a uniform K_D of 10 nM. Each point represents an average over all unique comparisons between 15 independent simulations and the error bars correspond to ≈ 95% confidence intervals. The black curve is calculated according to equation 12 (corresponding to "h") and the red curve is calculated using equations 13 and 14 (corresponding to "h_N"). The two distances are very similar, with differences in copy number (which are ignored by the definition of h) leading to slightly larger distances h_N.

Given that large complexes tend to be comparatively rare (see Figure 6 in the main text) and distinct between cells (see Figure 5C in the main text), it is useful to stratify complexes into abundance classes and calculate how the distance between a pair of cells varies as a function of abundance class. For two cells i and j at time t, and each complex of type k in either cell, we obtained the arithmetic average of its copy number as $\overline{N_k}(i,j) = \frac{1}{2}(N_k(i) + N_k(j))$. We then binned complexes according to their average copy number, using exponentially distributed bins (e.g. $0 < \overline{N_k}(i,j) \leq 1$, $1 < \overline{N_k}(i,j) \leq 2$, $2 < \overline{N_k}(i,j) \leq 4$, etc.) in order to account for the log-normal distribution of copy numbers present in the simulations. Within a given bin, we calculated the average value of h_N based on the complexes within that bin and plotted the resulting values against the average abundance in that bin, Figure 9. The similarity between cells increases dramatically the higher the abundance class over which the distance h_N is computed.

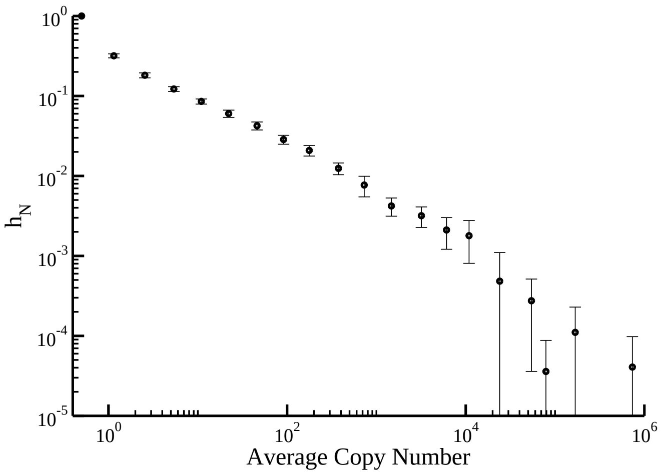

Figure 9. The dependence of h_N, equation 14, on the copy number of complexes. This figure is based on NR simulations with a uniform K_D of 10 nM and is computed at the last time point in Figure 5B in the main text. We calculated the values of h_N by first binning complexes according to their average copy number in two cells. The first bin, from 0 to 1, contains all of those complexes that have only one copy in one cell in a particular comparison. The bins are sized exponentially, with the upper endpoint of each bin separated from the upper endpoint of the preceding bin by a factor of 2. For each bin, we calculated the value of h_N for the complexes included in that bin. Each point represents an average over all unique comparisons between 15 independent simulations at the final time point of Figure 5B in the main text. The error bars correspond to $\approx 95\%$ confidence intervals. As copy numbers increase, the value of h_N decreases dramatically. The average distance varies approximately as a power law with an exponent of -0.7.

One can also consider the impact of copy numbers on the similarity between cells by computing the correlation between two cells as a function of complex size. The correlation is calculated in the natural way, by comparing the number of copies of any given complex in cell i to the number of copies of that same complex in cell j. As shown in Figure 10, small complexes exhibit a correlation very close to 1, indicating that the copy number of a small complex in one cell is a good predictor of the copy number of that complex in other cells of a population. This is mostly due to the fact that certain small complexes occur at very high numbers (see Figure 6 in the main text); these very common complexes tend to dominate the correlation. At intermediate sizes, some variation in copy number is observed, while for large complexes (i.e. complexes larger than 10), we obtain correlations ~ -1. This is due to the fact that cells do not exhibit much overlap in their repertoire of large complexes: if a complex is present in cell i, it is typically absent in j, and vice versa, leading to a correlation of -1.

Taken together, the above results indicate that the difference between cells depends strongly on the size of the complexes over which distance is computed. Small complexes, which tend to exist

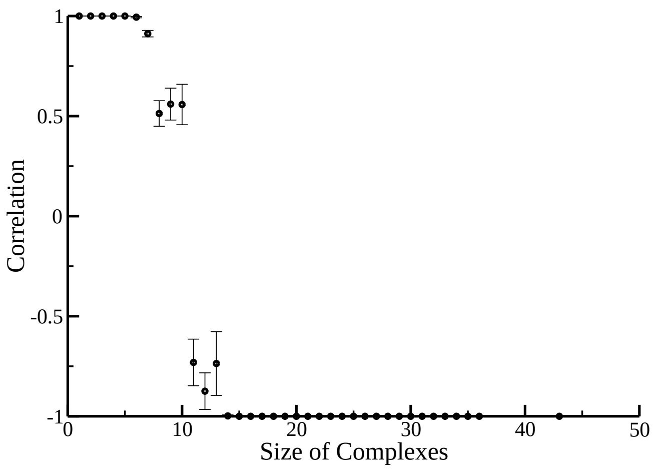

Figure 10. The correlation between two cells as a function of complex size. Each point in this plot represents an average over all unique pairwise comparisons between 15 independent simulations and the error bars correspond to ≈ 95% confidence intervals. In this case, the data is taken from NR simulations at a K_D of 10 nM; the comparisons are made between simulations at the final time point in Figure 5A of the main text. The correlation at each complex size is calculated by comparing the copy number of any given complex in a cell i to the copy number of that same complex in cell j and computing Pearson's correlation in the usual fashion. We observe correlations ~ 1 for small complexes while, for large complexes, we find a correlation of ~ -1. The perfect negative correlation at large complex sizes is due to the fact that, if a large complex is present in cell i, it will not be present in cell j, and vice versa.

at high copy numbers, do not vary significantly between cells in terms of their presence or absence (see Figure 5C in the main text), but do exhibit some differences in copy number (Figures 9 and 10). Complexes of intermediate size tend to have considerably smaller copy numbers and somewhat larger variation (either in presence/absence or in copy number) between cells. Individual large complexes tend to be found in very small copy numbers (on average just 1 copy per cell) and each large complex tends to be unique to a given cell. All other alternative definitions of distance that we have examined (including the simple Euclidean distance between the copy number vectors of two cells, the angle between those vectors, etc.) produce exactly the same results as those discussed here (data not shown).

8.2 Structure-based affinities

In the main text we consider three protocols for assigning affinities to the interactions listed in the cSIN. One protocol is based on estimating affinities from available crystal structures of domain-domain interactions. To identify such interactions and their corresponding structures, we

must construct *de novo* the cSIN, which we originally extracted from the SIN. We shall refer to the new version as the cSIN2.

The construction of the yeast SIN by Gerstein and coworkers [19] begins with a set of high-confidence interactions defined at the level of whole proteins (i.e. without information regarding sites). For each protein in this set, a domain structure is obtained based on the Pfam database of protein domains [20]. If two proteins interact according to the high-confidence list, the SIN construction checks whether any of their domains interact according to iPfam [21], a domain-domain interaction database derived from an analysis of solved protein structures. If a high-confidence interaction can be reconciled to a domain-domain interaction, the domains are added to the corresponding proteins in the SIN and edges are placed between them. These domains thus represent the "sites" of the SIN (and hence the cSIN), see Figure 2 of the main text.

We estimated the interaction affinity between any two domains in the cSIN on the basis of the protein structures used to define that particular domain-domain interaction in iPfam. To do this we first re-created the cSIN using an updated release of Pfam (we employed Pfam release 21, since this release contained the most recent version of iPfam at the time of this writing). We shall refer to this updated cSIN as cSIN2. We first determine the Pfam domains present in each protein of the original cSIN. We then determine for every interacting pair of proteins their corresponding set of interacting domains, as defined in iPfam. For example, suppose that proteins `A` and `B` interact in the cSIN and that domains {`k`,`l`,`m`} are found in `A` and domains {`m`,`n`,`o`} are found in `B`. If iPfam asserts that domains of type "`l`" are known to interact with domains of type "`m`", we add a site corresponding to domain `l` to protein `A` and a site corresponding to domain `m` to protein `B` and draw an edge between these domain instances in the cSIN2. Since an edge is placed between *all* sites that can interact, two proteins may have more than one edge connecting them. The cSIN2 obtained with this updated release of Pfam is very similar to the original cSIN (data not shown).

Every type of domain-domain interaction in iPfam is derived from at least one protein structure in which residues from the two domains are in close proximity to one another [21]. From that list of structures, we extracted a set of structures (PDB file names) representing every domain interaction occurring in the cSIN2. Given the thermodynamic differences governing intra- vs intermolecular interactions (see section 6.1 above), we removed from this set any structure in which the interaction domains are found on the *same* chain in the PDB file. We thus obtained a set of (co-crystal) structures containing all intermolecular instances of any domain-domain interaction ocurring in the cSIN2.

For each structure containing an intermolecular domain-domain interaction – say between domains `l` and `m` – we calculated the change in solvent-accessible, non-polar surface area ($\Delta\mathrm{SASA}_{\mathrm{NP}}$) using the software package POPS [22]. We proceeded by creating 3 separate PDB files: file #1 contains only the atoms (ATOM records) that belong to the residues of the first domain `l`, file #2 contains only the atoms that belong to the residues of the second domain `m`, and file #3 contains the atoms from both `l` and `m`. In cases with more than one copy of a particular chain in the coordinates (as is often the case with structures derived from NMR), we used the coordinates from the first chain mentioned in the PDB file. Next, we calculated the non-polar solvent-accessible surface area for each file separately using POPs. This area is marked as "hydrophobic" in the POPs output. We then calculated $\Delta\mathrm{SASA}_{NP}$ as the difference between

the sum of these areas for each domain separately and the area for the domains combined:

$$\Delta\text{SASA}_{\text{NP}}(\mathtt{l},\mathtt{m}) = \text{SASA}_{\text{NP}}(\mathtt{l}) + \text{SASA}_{\text{NP}}(\mathtt{m}) - \text{SASA}_{\text{NP}}(\mathtt{l}+\mathtt{m}). \quad (15)$$

We calculated $\Delta\text{SASA}_{\text{NP}}$ using definition 15 for all the intermolecular domain-domain interactions in the cSIN2.

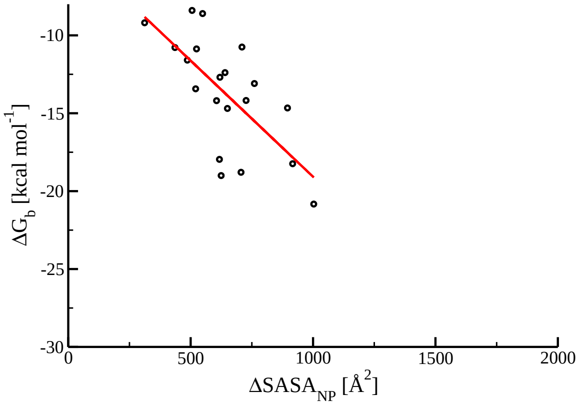

Figure 11. Solvent-accessible non-polar surface areas and free energies of binding. The graph depicts the relationship between the change in solvent-accessible non-polar surface area, $\Delta\text{SASA}_{\text{NP}}$ as defined in equation 15, and the free energy of binding. The free energies and the structures on which we based $\Delta\text{SASA}_{\text{NP}}$ were taken from Table 1 in [23] and $\Delta\text{SASA}_{\text{NP}}$ is calculated using POPS [22]. The red line represents a least-squares linear regression of the data, yielding equation 16 with $\text{R}^2 = 0.47$.

Several studies have noted that $\Delta\text{SASA}_{\text{NP}}$ is related to the free energy of binding ΔG_b [23, 24]. To map $\Delta\text{SASA}_{\text{NP}}$ into ΔG_b, we used a compilation of 20 individual structures that contain interactions whose ΔG_b had been measured [23]. In Figure 11, we plot the $\Delta\text{SASA}_{\text{NP}}$ of these structures, calculated using equation 15, against their measured ΔG_b. We found that the relationship between the free energy of binding and the associated change in solvent-accessible non-polar surace area is approximately linear, with an R^2 value of 0.47. We converted the $\Delta\text{SASA}_{\text{NP}}$ values obtained for the interactions in the cSIN2 using the best-fit linear equation:

$$\Delta G_b(\mathtt{l},\mathtt{m}) = -0.015 \cdot \Delta\text{SASA}_{\text{NP}}(\mathtt{l},\mathtt{m}) - 4.17 \quad (16)$$

with $\Delta\text{SASA}_{\text{NP}}(\mathtt{l},\mathtt{m})$ given in Å^2 and $\Delta G_b(\mathtt{l},\mathtt{n})$ in kcal mol^{-1}.

We used equation 16 to obtain binding free energies for the interactions in our cSIN2. In this process, we discarded any domain-domain interactions that buried less than 100 Å^2 of non-polar surface area, as they are unlikely to contribute strongly to the overall protein-protein interaction as captured in that particular structure. The binding free energy of the interaction between domains l and m is then set to be the average $\langle \Delta G_b(\text{l},\text{m}) \rangle$ of the free energies resulting from all

structures capturing the interaction between these domains and burying more than 100 $\mathring{A}^2$. Finally, the K_D for each interaction is defined as $K_D(\mathrm{l},\mathrm{m}) = \exp\left(\frac{\langle \Delta G_b(\mathrm{l},\mathrm{m}) \rangle}{RT}\right)$ with R the gas constant and T the absolute temperature. Here we consider interactions at room temperature, so RT is approximately 0.6 kcal mol^{-1}.

8.3 Results for SR simulations

SR simulations behave quite differently from NR simulations, even for the same underlying parameter values, i.e. all K_D values set to 10 nM. The results for SR simulations at this affinity are shown in Figure 12, which is essentially analogous to Figure 5 (computed for the NR scenario) in the main text.

The SR constraint leads to considerably fewer unique molecular species at equilibrium (Figure 12a) when compared to the NR simulations ($\sim$ 2000 for the SR case vs. $\sim$ 10000 for the NR case, see Figure 5A in the main text). The system also takes much longer to reach equilibrium; the number of unique species initially climbs to a maximum value of $\sim$ 3000 but then decays slowly, reaching an apparent steady state after about 100 time units (compared to the $\sim$ 8 time units for the similar decay in the NR case, see Figure 5A in the main text). NR simulations that are run for the same length of time do not show any further signs of decay (data not shown). The SR scenario exhibits a more pronounced maximum because acyclic complexes that are formed initially eventually dissociate and are replaced by cyclic complexes, which are infinitely stable, resulting in a net decrease in the number of unique complexes on long time scales. Given the computational cost of running simulations that are this long (amounting to several weeks of CPU time), we cannot currently explore dynamics on a time scale longer than that shown in Figure 12a, which prevents us from determining whether the decay continues at a slow pace or has really ceased by $\sim$ 100 time units.

Figure 12b represents the distribution of complex sizes at the final time point in Figure 12a, analogous to Figure 6 in the main text for the NR scenario. Comparison of these figures reveals that SR simulations tend to sample much smaller complexes than NR simulations. SR simulations also tend to display less overall diversity; on long time scales the average distance between independent simulations is $\sim$ 0.25 (Figure 12c), as compared to $\sim$ 0.7 for the NR case (see Figure 5B in the main text). As can be seen from Figure 12d, however, SR simulations still exhibit significant diversity for large complexes. Indeed, the shape of this curve is essentially identical to that obtained in NR simulations (Figure 5C in the main text). The smaller overall distance between SR simulations is due to the fact that these simulations sample substantially fewer large complexes (Figure 12b), and does not indicate a significantly greater agreement between simulations in terms of which large complexes they contain. In fact, SR simulations tend to drift in the space of large complexes, much like NR simulations do (data not shown).

8.4 Results for different affinity scenarios

In this section we present an overview of the results obtained for various interaction affinity scenarios in the context of both the SR and NR scenarios. The results shown and discussed in the

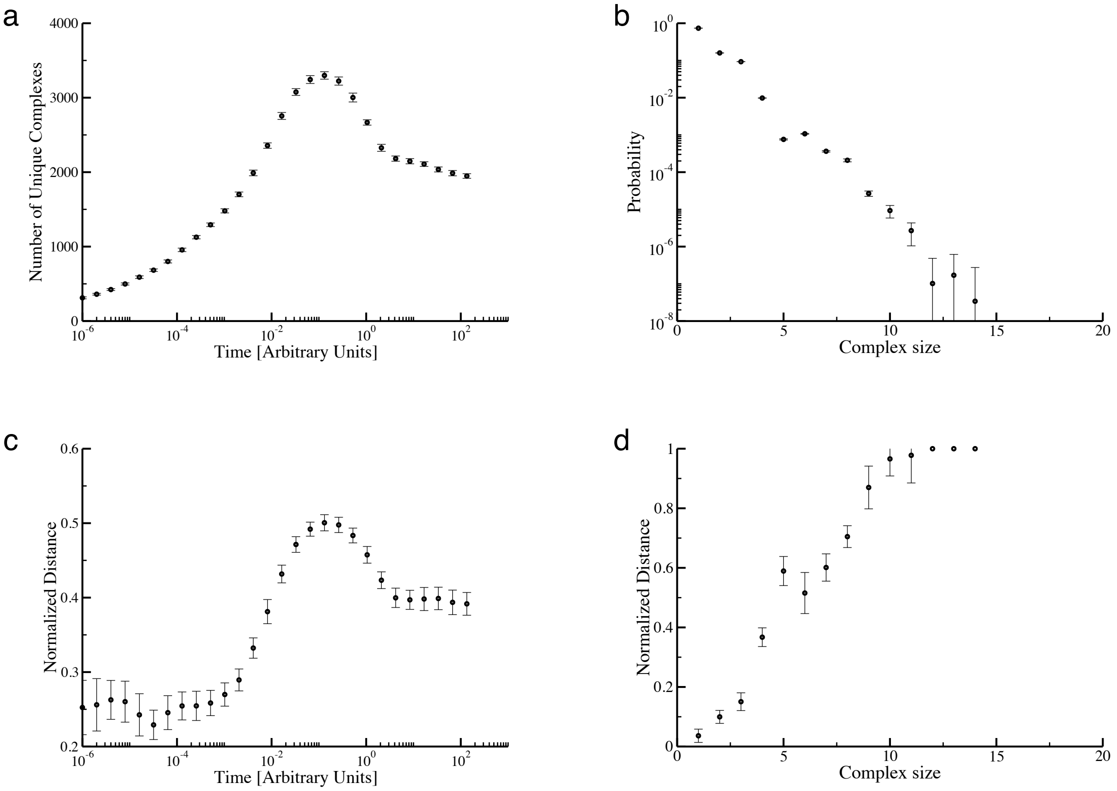

Figure 12. Simulations with the SR constraint. Results for simulations conducted using the SR constraints with all affinities set to $K_D = 10$ nM. **(a)** This curve plots the average number of unique molecular species (averaged across 15 independent simulations) as a function of time. The error bars in this and all of the other panels in this figure represent $\approx 95\%$ confidence intervals. The SR constraint clearly leads to many fewer unique complexes at steady state than the NR case. **(b)** The data points represent the average probability of finding a complex of a specified size across the entire population of 15 simulations at the final time point in panel **a**. The NR simulations have a much higher probability of sampling large complexes (i.e. complexes of size > 7) than the SR simulations shown here. **(c)** This curve represents the distance between independent simulations, averaged across all unique comparisons between 5 simulations. As in panel **a**, the simulations exhibit a maximum distance (~ 0.33) before relaxing to a steady-state value of ~ 0.25. The distances observed between simulations are much smaller for the SR case than the NR case (compare with Figure 5B in the main text). **(d)** This curve represents the distance between simulations as a function of complex size, averaged over 5 simulations for the final time point in panel **a**. SR simulations show significant distances for large complexes. Taken together, this figure and panel **b** indicate that SR simulations exhibit smaller overall distances than NR simulations at steady state not because they exhibit greater similarity in their large complexes, but simply because they tend to sample much smaller complexes than NR simulations.

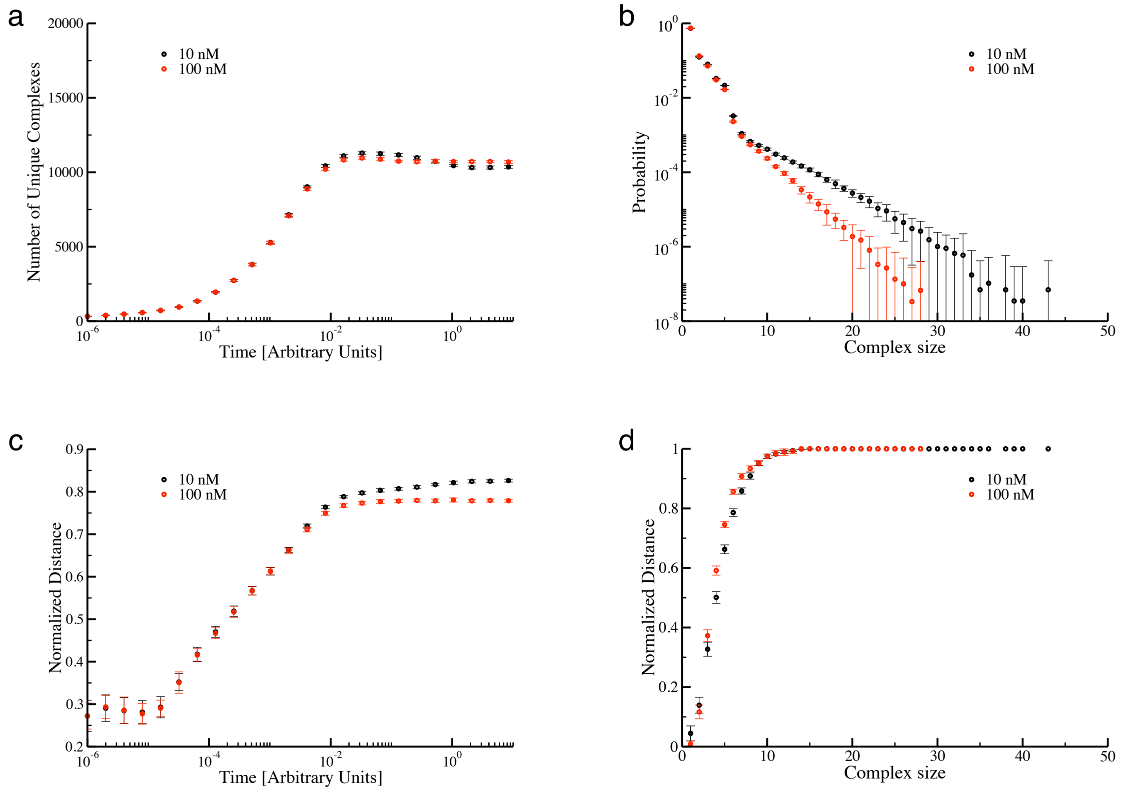

Figure 13. Comparison of the 10 nM **and** 100 nM **interaction affinity scenarios (NR constraint). (a)** Each curve represents the average number of unique complexes as a function of time (averaged over 15 independent simulations). The error bars in all panels in this figure represent ≈ 95% confidence intervals. Weakening the interaction affinity by one order does not to strongly influence the steady state; the average number of unique complexes is essentially identical for the two cases. The 100 nM clearly lack the slight peak in the number of unique species observed in the 10 nM and actually exhibit slightly larger numbers of unique species at steady state. **(b)** Comparison of the distribution of complex sizes: the distributions represent the average probability of finding a complex of a particular size across the entire population of 15 simulations at the final time point in panel **a**. Networks with dissociation constant 10 nM have a higher probability of sampling large complexes (i.e. complexes of size > 7) than those with dissociation constant 100 nM. **(c)** Comparison of the distance between independent simulations over time: each curve represents the average over all unique comparisons between 15 independent simulations using the distance measure defined in the main text. As in panel **a**, both interaction affinity scenarios produce strikingly similar curves; at steady state, the average normalized distance between $K_D = 10$ nM simulations is ~ 0.83 and for $K_D = 100$ nM the average distance is ~ 0.78. **(d)** Comparison of the distance between independent simulations as a function of complex size: each curve represents the average over all unique comparisons between 15 independent simulations at the final time point in panel **a**. Again, the two affinity scenarios show very similar behavior; the major difference is that the 10 nM simulations sample much larger complexes than the 100 nM simulations.

main text are all obtained from NR simulations in which all affinities were set to $K_D = 10$ nM. Figure 13 compares the 10 nM results to the 100 nM results for NR simulations; this figure is essentially analogous to Figure 5 in the main text.

One finds that weaker interactions generally lead to smaller complexes (Figure 13b). This result is not surprising given that lower affinities give rise to fewer bonds and therefore smaller complexes in general. Since there are fewer unique options for smaller complexes (see Figure 4 in the main text), between-simulation distances are accordingly smaller (Figure 13c). As with the SR case at 10 nM, this is *not* due to the fact that simulations are *more* similar to each other with regard to the large complexes they contain, as demonstrated in Figure 13d. The smaller distances result from the fact that each simulation samples fewer large complexes, resulting in smaller overall distances even though the sets of large complexes in each simulation are essentially disjoint. We find the same general behavior for simulations at $K_D = 1$ μM. Such a low affinity sustains even fewer bonds, and thus even smaller complexes and inter-simulation distances (data not shown). Yet, even at this affinity, simulations disagree with regard to the large complexes they sample (data not shown).

8.5 Results based on adding "cryptic" cycles

The thermodynamics of ring-like protein complexes (section 6.1) can result in situations in which a particular pair of sites might not bind one another strongly enough to be detected in a high-throughput interaction screen but could nonetheless contribute dramatically to the stability of certain complexes by forming a bond to complete a ring. Here we consider how such "cryptic" cycles (i.e. cycles that do not exist in the contact map but that may form very stable ring-like structures in a complex) influence the dynamics of our simulations.

By definition, the cSIN does not directly contain any information regarding cryptic cycles. In the absence of data on which proteins could form cryptic cycles, we add these cycles randomly to the graph and assess how increasing their number influences the behavior of the system. Given the computational cost of simulating the entire cSIN, we restricted this analysis just to the giant component of the cSIN (i.e. the cluster of proteins in the upper right-hand corner of Figure 2 in the main text). This allowed us to perform many simulations with varying amounts of cryptic cycles. For simplicity, we focused our analysis on cryptic cycles of length 3 (i.e. cycles containing only three proteins).

We first collected the set of protein triples that do not belong to a proper cycle in the giant component of the cSIN but could form a proper cycle if one edge were added to the graph. This set includes all proteins "`A`", "`B`" and "`C`" such that (i) `A`, `B` and `C` are not part of a proper cycle in the cSIN, (ii) `A` can bind both `B` and `C` simultaneously, and (iii) both `B` and `C` have at least two sites. In the giant component of the cSIN we identified 113 distinct sets of proteins meeting the criterion of a "potential" cryptic cycle. We then created new versions of the giant component of the cSIN in which a fraction (20%, 30%, etc.) of these potential cycles were converted into proper cycles by adding a corresponding binding rule to the system. Given the underlying hypothesis of the cryptic cycle (i.e. that binding between `B` and `C` is not strong enough to occur appreciably on its own), we added these new rules with very low on-rates ($\beta_+ = 10^{-8}$). In SR simulations cryptic cycles will still form infinitely stable ring-like structures despite the low on-rate (low affinity) of

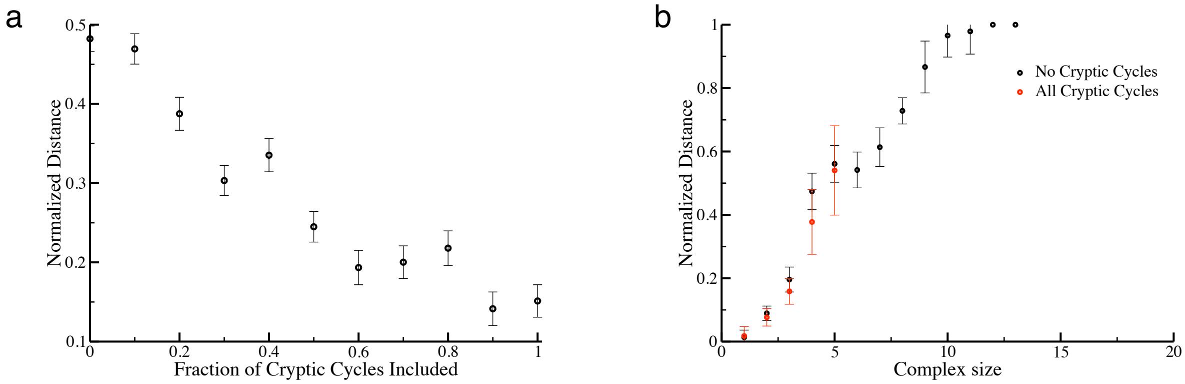

Figure 14. Cryptic cycles. Results based on simulations of the giant component of the cSIN in which cryptic cycles have been added to the graph. **(a)** The plot shows the average distance d for simulations of networks with a varying fraction of cryptic cycles added to the giant component of the cSIN, as described in the text. The "0" point corresponds to a graph in which no cryptic cycles are added (i.e. the giant component of the cSIN), while the point at a fraction of "1" represents a case in which all possible 113 cryptic cycles were added to the graph. The simulations were performed using the SR constraint and a constant K_D of 10 nM for all original reactions in the network (i.e. *not* the reactions added to generate cryptic cycles). Each point represents the average distance over all unique comparisons between 10 independent simulations at a particular time t after the simulations reach steady state (taken to be ~ 30 time units, see Figure 12c). The error bars represent $\approx 95\%$ confidence intervals. The average steady-state distance decreases quite dramatically as the number of cryptic cycles increases. **(b)** Here we plot the distance between complexes as a function of complex size for simulations with "No Cryptic Cycles" (the point corresponding to a value of 0 on the abscissa of panel **a**) and for simulations including "All Cryptic Cycles" (corresponding to the point at 1 in panel **a**). As one can see, adding cryptic cycles essentially reduces the maximum complex size observed in the simulation (in this case from 13 with no cycles to 5 when all possible cycles are included). The reduction in overall distance observed at higher cryptic cycle densities results from these cycles effectively preventing the sampling of large complexes.

the `B-C` bond (see section 6.2).

We constructed a set of graphs with a fraction of cryptic cycles ranging from 10% to 100%. In each graph, the set of cryptic cycles added to the system was chosen uniformly at random from the set of 113 possibilities. If more than one pair of sites could be used to generate a proper cycle from a given potential cycle, we chose one such pair at random with equal probability from the set of possible pairs. We ran 10 independent simulations for each graph and calculated the average steady-state distance between simulations (using distance "d" from equation 11) as a function of the fraction of cryptic cycles included in the graph. The results are shown in Figure 14.

Adding cryptic cycles to the graph clearly decreases the overall distance between simulations at steady state. Including all of the possible cycles (corresponding to a fraction of 1 in Figure 14a) results in a distance of only 0.15 compared to a distance of 0.5 when no cryptic cycles are added. The addition of cryptic cycles results in simulations that tend to sample much smaller complexes: when all cryptic cycles are added, the largest complex formed by the system has only 5 members, compared to 13 when no cryptic cycles are present. The net effect of including cryptic cycles is much like the effect of including the SR (vs. NR) constraint; the overall distance between cells decreases not because cryptic cycles increase the similarity between large complexes but rather because cryptic cycles dramatically reduce the size of complexes sampled by the simulation.

9 Comparison with Affinity Purification / Mass Spectrometry data

In this section we aim at validating the structure of the cSIN (beyond its original curation by Kim *et al* [19]) and the soundness of our dynamical process (including global constraints) by comparing simulation outcomes with available data germane to complex formation in yeast cells. At present, there is no direct experimental method that can determine all of the individual complexes present in a single cell at a given time, but we can compare simulation outcomes to Affinity Purification-Mass Spectrometry (AP-MS) data [25, 26]. This type of data samples complexes from a large number of cells and provides only limited insight into the detailed structure of the complexes themselves. Although one can attempt to reconstruct sets of complexes from this data (e.g. [26, 27]), many different sets of putative complexes can be equally consistent with a given set of empirical results.

AP-MS experiments ultimately produce putative co-complex relationships [27]. Our simulations produce a set of structured complexes that we can convert into a co-complex relationship of the AP-MS kind by defining two proteins A and B to be related if they are found together in at least one complex in a population of simulations at a particular time t. We create our set of co-complex relationships by examining the 15 independent simulations for a given set of conditions (either the SR or NR scenarios at $K_D = 10$ nM) at a particular time point after steady-state in the number of unique complexes per cell has been reached (~ 8 time units for the NR scenario and ~ 100 time units for the SR scenario).

We then compare the set of simulated co-complex interactions to the set observed in a recent collation of AP-MS data employed by Yu et al. [25]. This data was downloaded directly from the authors' website at http://interactome.dfci.harvard.edu/S_cerevisiae. We calculate a parameter f that represents the fraction of interactions observed in the data that are also observed in our simulation. Naturally, f is restricted to the set of interactions between proteins that are included in the cSIN, which yields a total of 48 co-complex interactions in the data. Of these 48 interactions, however, 11 occur between pairs of proteins that belong to different components in the cSIN (i.e. are found in different clusters in Figure 2 of the main text). Since there is no path in the graph linking the two proteins in these 11 pairs, our simulation cannot produce co-complex interactions for any of them.

As discussed in the main text, the cSIN is derived from the giant component of the SIN, indicating that the underlying PPI data contains a path connecting each pair in this set of 11. In most cases, these paths are missing from the cSIN due to the fact that proteins on this path had either

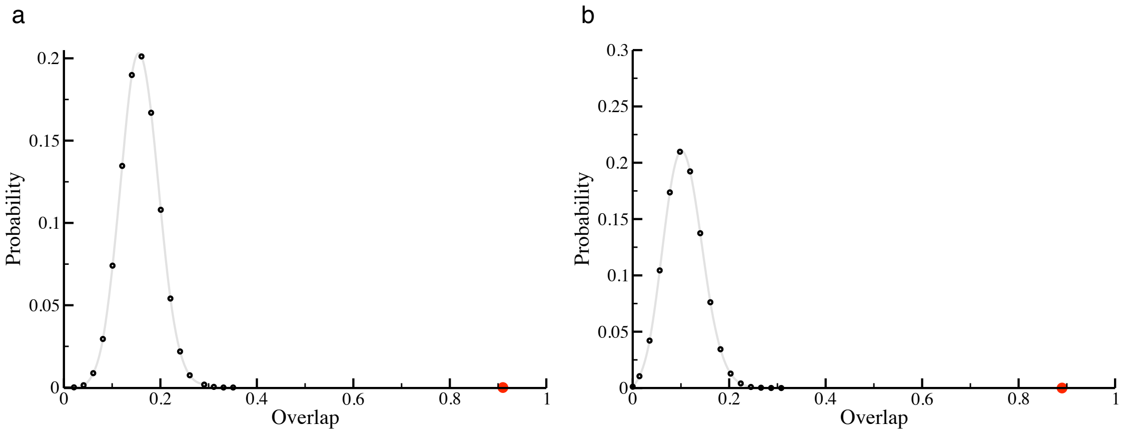

Figure 15. Overlap with AP-MS data. Distribution of the overlap between randomized SR co-complex interactions and the AP-MS data from [25]. Panel (b): Simulations with the SR scenario. In both of these plots we compare the outcomes of our simulations with AP-MS data. The abscissa indicates the overlap between simulation data and co-complex interactions observed by AP-MS. The red dot marks the overlap between our simulations and experimental data. The black data points represent a histogram of the frequency with which a particular overlap is observed in a set of 100,000 randomizations of our simulation data ("randomized overlap"), as described in the text. The grey curve represents a smoothed version of this distribution. The separation between the randomized overlap and the actual overlap indicates that our simulation outcomes are extremely significant. Note: This plot is based on a limited set of interactions (only 48 interactions involving cSIN proteins are actually covered in the AP-MS data). Panel (a): Simulations with the NR scenario.

"ambiguous" localizations or were simply not visualized in the experimental localization data [28]. In a few cases, a path exists between the two proteins in question in a different compartment (such as the nucleus). Since AP-MS experiments assay all of the compartments in the cell, they recover co-complex interactions that we cannot observe in simulations of the cytoplasm.

If we restrict our comparison only to those interactions that do not suffer from the localization issues discussed above, we obtain an f of 91% for the NR simulations and 89% for the SR simulations. Considering all of the 48 co-complex interaction possibilities reduces the overlap to 71% and 69%, respectively.

To understand whether the degree of overlap we observe could be explained by random association of proteins in a co-complex graph, we performed a randomization of each set of simulated co-complex interactions by randomly swapping interaction pairs (essentially randomly rewiring the co-complex graph). For both the SR and the NR scenario, we performed 10^5 such randomizations. The comparison between the distribution of f-values in these randomized sets and the actual simulations is shown in Figure 15 for the NR and SR scenarios. In both scenarios we see that the p-value of observing the values of f that we see is $\ll 10^{-5}$; this is true regardless of whether we consider the simulation overlaps to be $\sim 70\%$ rather than $\sim 90\%$. We have also compared our results to the set of "core" complexes observed by Gavin et al. [26], with essentially

identical results to those shown above (data not shown).

We found that the level of overlap for NR and SR simulations did not vary with interaction strength, nor did either case produce a different overlap with experiment when affinities were determined using the concentration-based scenario (derived from equation 4 in the main text). The cSIN2, however, results in only a 54% overlap with AP-MS results. This is because a number of edges in the cSIN are missing from the cSIN2, as suitable structures to define the affinities between certain types of domain-domain interactions could not be found. These edges evidently carry significant levels of information from the standpoint of complex formation (given the much lower overlap between cSIN2 simulations and the experimental results). For this reason we focus on the 10 nM results in the main text, although the overall behavior of the cSIN2 is very similar to the constant-affinity case of the original cSIN (see Figure 9 in the main text).

It is important to note two facts about this comparison. First, the AP-MS data from Yu et al. contain only a small number of distinct co-complex interactions when restricted to cSIN proteins. Indeed, about 50% of the proteins included in our simulations are not mentioned in this data set at all. Thus, the values of f calculated above, while quite statistically significant, are based on a comparatively small number of data points. Second, our simulations predict hundreds of co-complex associations that are not observed, yet *could* be detectable in principle (because they occur between two proteins that are both mentioned in the AP-MS dataset; for which, therefore, some number of co-complexes have been identified). Many such interactions are actually purely binary interactions observed in highly curated datasets (including the SIN) yet are not found in the AP-MS data [25]. Either these interactions do not exist in cells (and thus represent errors in our approach) or they do exist but have not been observed (and thus represent errors or omissions in the AP-MS data).

References

1. Blinov ML, Faeder JR, Hlavacek WS (2004) BioNetGen: Software for rule-based modeling of signal transduction based on the interactions of molecular domains. Bioinformatics 20: 3289-3292.

2. Danos V, Laneve C (2004) Formal molecular biology. Theoretical Computer Science 325: 69–110.

3. Lok L, Brent R (2005) Automatic generation of cellular reaction networks with moleculizer 1.0. Nature Biotechnology 23: 131–136.

4. Danos V, Feret J, Fontana W, Harmer R, Krivine J (2007) Rule-based modelling of cellular signalling. In: Proceedings of the 18th Int. Conf. on Concurrency Theory. Lisboa, Portugal: Springer, volume 4703 of *Lecture Notes in Computer Science*, pp. 17–41.

5. Mallavarapu A, Thomson M, Ullian B, Gunawardena J (2008) Programming with models: modularity and abstraction provide powerful capabilities for systems biology. J Roy Soc Interface doi:10.1098/rsif.2008.0205.

6. Hlavacek WS, Faeder JR, Blinov ML, Posner RG, Hucka M, et al. (2006) Rules for modeling signal-transduction systems. Science STKE 344: re6.

7. Feret J, Danos V, Krivine J, Harmer R, Fontana W (2009) Internal coarse-graining of molecular systems. Proc Natl Acad Sci USA 106: 6453-6458.

8. Harmer R, Danos V, Feret J, Krivine J, Fontana W (2010) Intrinsic information carriers in combinatorial dynamical systems. Chaos 20: 037108.

9. Doob JL (1945) Markoff chains denumerable case. Trans Amer Math Soc 58: 455-473.

10. Gillespie DT (1976) A general method for numerically simulating the stochastic time evolution of coupled chemical reactions. Journal of Computational Physics 22: 403–434.

11. Danos V, Feret J, Fontana W, Krivine J (2007) Scalable simulation of cellular signalling networks. In: Proceedings APLAS 2007. Springer, volume 4807 of *Lecture Notes in Computer Science*, pp. 139–157.

12. Yang J, Monine MI, Faeder JR, Hlavacek WS (2008) Kinetic monte carlo method for rule-based modeling of biochemical networks. Phys Rev E 78: 031910.

13. Danos V, Feret J, Fontana W, Krivine J (2008) Abstract interpretation of cellular signalling networks. In: Verification, Model Checking, and Abstract Interpretation. Springer, volume 4905 of *Lecture Notes in Computer Science*, pp. 83–97.

14. Wolfram Research, Inc (2008) Mathematica Edition: Version 7.0. Champaign, Illinois: Wolfram Research, Inc.

15. Saiz L, Vilar JM (2006) Stochastic dynamics of macromolecular-assembly networks. Mol Syst Biol 2: 2006 0024.

16. Minh DD, Bui JM, Chang CE, Jain T, Swanson JM, et al. (2005) The entropic cost of protein-protein association: a case study on acetylcholinesterase binding to fasciculin-2. Biophys J 89: L25–7.

17. Bray D, Lay S (1997) Computer-based analysis of the binding steps in protein complex formation. Proc Natl Acad Sci U S A 94: 13493–8.

18. Jorgensen P, Nishikawa JL, Breitkreutz BJ, Tyers M (2002) Systematic identification of pathways that couple cell growth and division in yeast. Science 297: 395–400.

19. Kim PM, Lu LJ, Xia Y, Gerstein MB (2006) Relating three-dimensional structures to protein networks provides evolutionary insights. Science 314: 1938–41.

20. Finn RD, Tate J, Mistry J, Coggill PC, Sammut SJ, et al. (2008) The pfam protein families database. Nucleic Acids Res 36: D281–8.

21. Finn R, Marshall M, Bateman A (2004) ipfam: visualization of protein-protein interactions in pdb at domain and amino acid resolutions. Bioinformatics 21: 410-412.

22. Fraternali F, Cavallo L (2002) Parameter optimized surfaces (pops): analysis of key interactions and conformational changes in the ribosome. Nucleic Acids Res 30: 2950-2960.

23. Bougouffa S, Warwicker J (2008) Volume-based solvation models out-perform area-based models in combined studies of wild-type and mutated protein-protein interfaces. BMC Bioinformatics 9: 448.

24. Horton N, Lewis M (1992) Calculation of the free energy of association for protein complexes. Protein Sci 1: 169-81.

25. Yu H, Braun P, Yildirim MA, Lemmens I, Venkatesan K, et al. (2008) High-quality binary protein interaction map of the yeast interactome network. Science 322: 104–110.

26. Gavin AC, Aloy P, Grandi P, Krause R, Boesche M, et al. (2006) Proteome survey reveals modularity of the yeast cell machinery. Nature 440: 631–636.

27. Scholtens D, Gentleman R (2004) Making sense of high-throughput protein-protein interaction data. Stat Appl Genet Mol Biol 3: Article39.

28. Huh WK, Falvo JV, Gerke LC, Carroll AS, Howson RW, et al. (2003) Global analysis of protein localization in budding yeast. Nature 425: 686–91.
